# Cell stimulation versus cell death induced by sequential treatments with pulsed electric fields and cold atmospheric pressure plasma

**DOI:** 10.1371/journal.pone.0204916

**Published:** 2018-10-12

**Authors:** Anna Steuer, Christina M. Wolff, Thomas von Woedtke, Klaus-Dieter Weltmann, Juergen F. Kolb

**Affiliations:** 1 Leibniz Institute for Plasma Science and Technology (INP Greifswald), Greifswald, Germany; 2 Centre for Innovation Competence (ZIK) *plasmatis*, Greifswald, Germany; Universita degli Studi di Palermo, ITALY

## Abstract

Pulsed electric fields (PEFs) and cold atmospheric pressure plasma (CAP) are currently both investigated for medical applications. The exposure of cells to PEFs can induce the formation of pores in cell membranes and consequently facilitate the uptake of molecules. In contrast, CAP mainly acts through reactive species that are generated in the liquid environment. The objective of this study was to determine, if PEFs combined with plasma-treated cell culture medium can mutually reinforce effects on viability of mammalian cells. Experiments were conducted with rat liver epithelial WB-F344 cells and their tumorigenic counterpart WB-ras for a direct comparison of non-tumorigenic and tumorigenic cells from the same origin. Viability after treatments strongly depended on cell type and applied field strength. Notably, tumorigenic WB-ras cells responded more sensitive to the respective treatments than non-tumorigenic WB-F344 cells. More cells were killed when plasma-treated medium was applied first in combination with treatments with 100-μs PEFs. For the reversed treatment order, i.e. application of PEFs first, the combination with 100-ns PEFs resulted in a stimulating effect for non-tumorigenic but not for tumorigenic cells. The results suggest that other mechanisms, besides simple pore formation, contributed to the mutually reinforcing effects of the two methods.

## Introduction

Pulsed electric fields (PEFs) with pulse durations in the range of microseconds to milliseconds can lead to the formation of pores in the cell membrane, when the induced transmembrane potential exceeds a certain threshold, generally in the order of 1 V. Pores that are created facilitate the influx of ions and molecules. This principle is the basis for electrochemotherapy (ECT) where large, hydrophilic cytostatic drug molecules, that normally poorly enter the cell, can be taken up by the cell more easily [1]. ECT is in the meantime an established treatment option in clinics, in particular for patients suffering from end-stage melanoma [2–8].

PEFs alone, i.e. without a combination with cytostatic drugs, and in particular nanosecond PEFs (nsPEFs), are currently investigated for their potential for cancer treatment. Different studies showed a PEF-induced caspase-dependent and -independent induction of apoptosis in cancer cells, DNA fragmentation, a decrease of the mitochondrial membrane potential and an increase of the intracellular calcium level [9–18]. An antitumor effect could also be demonstrated in several animal studies leading to a complete tumor remission, or at least a tumor volume reduction as well as a disruption of the tumor´s blood supply [10, 19–26]. The induction of apoptosis was also demonstrated *in vitro* as well as *in vivo* for longer pulses with a duration in the range of microseconds [9, 20, 27, 28].

Cold atmospheric pressure plasma (CAP) has likewise shown potential for cell manipulation and medical applications. Depending on the plasma treatment time, different effects were observed when CAP was applied to cells or tissue. For relatively short treatment times of 1–2 min, proliferation and angiogenesis are stimulated [29–31]. Conversely, longer plasma treatment times can result in the induction of apoptosis [32–34]. The interaction of plasma with cells is mediated in particular by reactive species that are formed in aqueous solutions including cell environments, e.g. media or extracellular fluids, when exposed to CAP. Respective nitrogen and oxygen species target for example oxidable membrane lipids and enzymes [35–38]. Therefore, a direct plasma-exposure does in fact not seem to be necessary to cause an effect on cells.

It seems reasonable to assume that PEFs can facilitate the uptake of plasma-generated reactive species and thus enhance effects of CAP-exposures. Killing but also stimulation has been reported for short plasma treatment times [32, 33]. A first study on the combined treatment of CAP together with PEFs was already conducted by Zhang et al. who investigated the viability of the bacteria strain *Staphylococcus aureus*. They found a synergistic antibacterial effect which was dependent on the treatment order [39]. The effect was merely additive when PEFs were applied first but synergistic when bacteria were treated with CAP first. Another study by Daeschlein et al. investigated a possible antitumor efficacy of CAP in a melanoma mouse model in comparison with ECT only [40]. The cytostatic drug bleomycin was injected into the orbital vein of mice 4 min before the tumors were treated with either PEFs or CAP directly. A significant increase in survival-days after treatment was found for conventional ECT as well as for ECT combined with CAP, but not for the combination of PEFs with CAP without the use of a cytostatic drug. Not surprisingly, this confirms an effective killing of cancer cells by the cytostatic drug but does not suggest possible mechanisms between CAP and PEFs.

The objective of the work presented here was to determine if the two methods, PEFs and CAP, can reinforce each other and thus enhance responses of mammalian cells. Furthermore, it was investigated if tumor cells and non-tumorigenic cells respond differently to the same exposure conditions. Experiments were conducted on monolayers of WB-F344 and WB-ras cells, which are not only syngeneic (comparable to cells in primary tumors *in vivo*) but also known for their strong gap junctional intercellular communication *in vitro*. Thus, they serve as a simple tissue model that better represents the situation *in vivo* than suspended cells. It is hypothesized that the effect of plasma is mediated by reactive species generated in the liquid environment. Accordingly, cells were incubated in plasma-treated medium (PTM), avoiding a desiccation of cells that is associated with direct plasma treatment. Effects of the different treatments on cell viability were determined by an MTT assay which, compared to other live/dead assays, has the advantage that the respiratory activity is measured rather than the viability. Therefore, not only the killing but also a metabolic activation of cells could be detected.

## Materials & methods

### Cell culture

The rat liver epithelial cell line WB-F344 and its tumorigenic counterpart WB-ras were chosen for this study [41]. Both were obtained from J. E. Trosko, Michigan State University, East Lansing, MI, USA. WB-ras cells are WB-F344 cells, which were transfected with the oncogene *ras*, enabling to compare the response of similar cell types with either normal or tumorigenic characteristics. Cells were cultured in low-glucose DMEM (#P04-01500), supplemented with 2 mM L-glutamine, 5% fetal calf serum and 1% penicillin/streptomycin (#P06-07300) (all purchased from PAN-Biotech GmbH, Aidenbach, Germany) in a humidified CO_2_ (5%) incubator. A number of 4∙10^5^ cells were seeded in each well of a 12-well plate and incubated for 48 h to form confluent monolayers.

The human skin cell lines HaCaT and SK-MEL-28 were cultured in RPMI (#P04-17500, PAN-Biotech), supplemented with 2 mM L-glutamine, 8% fetal calf serum and 1% penicillin/streptomycin. Cells were seeded in 12-well plates and grown until a confluent monolayer was formed to conduct the experiments.

### Pulsed electric field exposures

**Fig 1:**
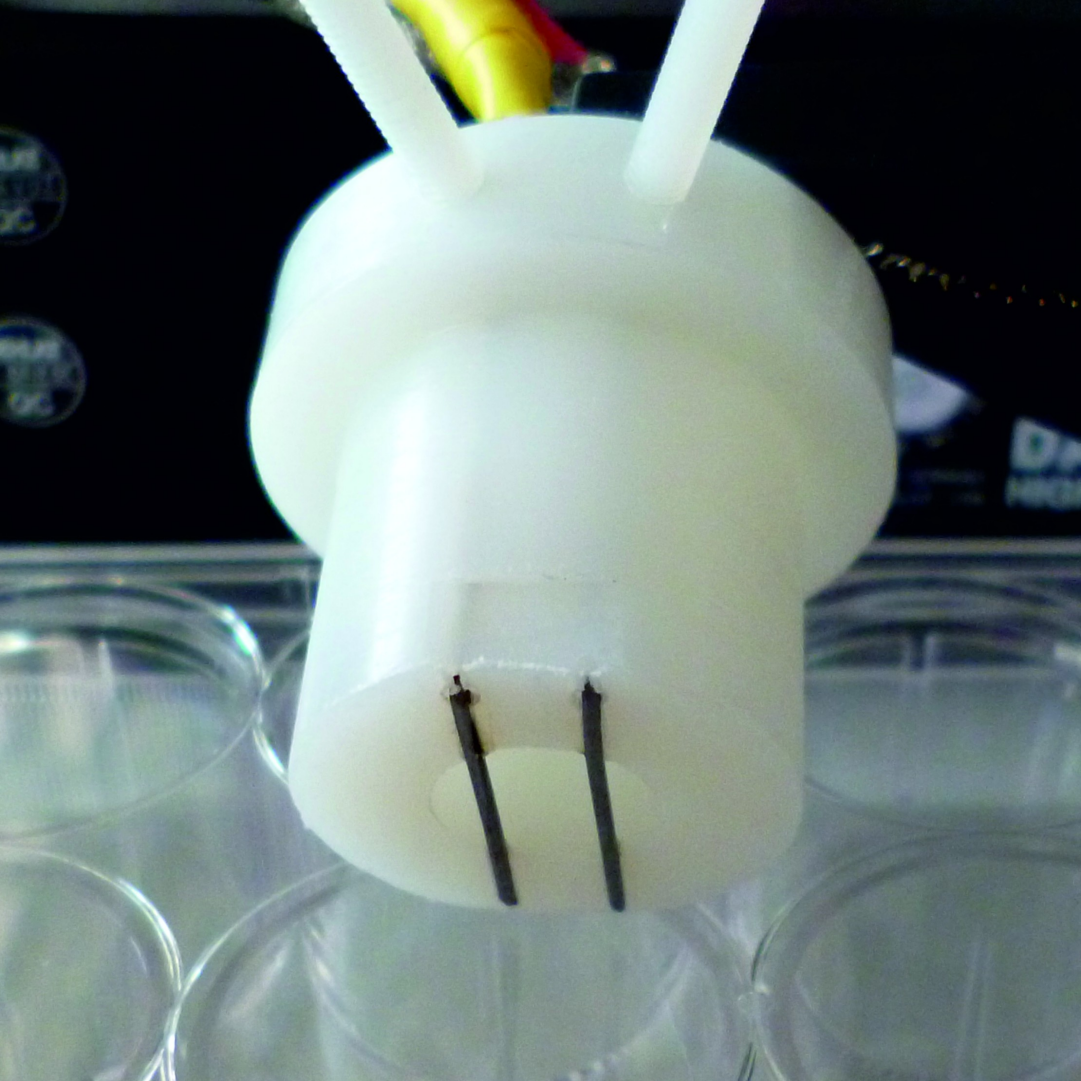
In-house built electrode configuration for the application of PEFs to a monolayer.

For the generation of microsecond pulsed electric fields (μsPEFs) a commercially available pulse generator (ECM 830, Harvard Apparatus, Holliston, MA, USA) was used. The electric pulses were applied by means of an in-house built electrode configuration (Fig 1), which consisted of two parallel stainless steel wires with a diameter of 0.8 mm and a center-to-center gap distance of 5 mm. The electrodes were fixed in a plastic cylinder that tightly fitted into individual wells of a 12-well plate. The electrodes were slightly impressed into the monolayer that was covered with cell culture medium and 8 pulses of 100 μs with amplitudes between 1.0 kV/cm and 1.5 kV/cm were applied. The parameters were chosen, because they are typically used for electroporation and in particular for electrochemotherapy [42].

NsPEFs were generated with an in-house built Blumlein line pulse generator which provided high voltage pulses with a duration of 100 ns. Twenty consecutive pulses with field strengths of 15 kV/cm, 20 kV/cm and 25 kV/cm were applied with the same electrode system that was used for μsPEFs-exposures. Pulse duration and field strengths have been repeatedly used for the treatment of tumors in animal studies [24, 26, 43, 44].

### Preparation of plasma treated medium

For the treatment of the medium that was administered to the monolayer, the well-studied kINPen 09 was used, developed at the INP Greifswald [45, 46]. The kINPen is a RF-driven plasma jet (6 kVpp, 1.1 MHz) that was operated with 5 slm argon gas. A volume of 5 ml of DMEM cell culture medium in a 60-mm Petri dish was exposed to plasma for 1, 2, 3 or 5 minutes. The original cell culture medium in the wells was replaced with 850 μl of the plasma treated medium (PTM) for the study of the effect on the cells. PTM was exclusively prepared from DMEM, regardless if the cells were grown in DMEM or RPMI before.

### Combined treatment

*Pulsed electric field treatment followed by exposure to plasma treated medium (1*. *PEF 2*. *PTM)*: After cells were exposed to PEFs, medium was removed from the cells and replaced by PTM within 2 min. PTM was kept on the cells until an MTT assay was performed.

*Exposure to plasma treated medium followed by pulsed electric field treatment (1*. *PTM 2*. *PEF)*: For the reversed treatment order, the medium on the cells was replaced by PTM and PEFs were applied within 2 min after the medium was exchanged. PTM was then kept on the cells until an MTT assay was performed. The medium on the control cells was also exchanged by fresh untreated medium (DMEM), before cells were incubated again until further processing.

### MTT assay

Effects on cells were determined by an MTT assay that quantifies the respiratory activity of cells, which is associated with their viability. Accordingly, not only a reduction but also an activation of cell metabolic activity can be detected. Cells were incubated for 3 h or 24 h after treatment before cell culture medium was replaced by 500 μl medium containing 50 μl MTT-solution (5 mg/ml in PBS; AppliChem, Darmstadt, Germany). MTT-solution was removed and the monolayers were rinsed once with Hank´s Balanced Salt Solution (HBSS) after 2 h of incubation at 37°C. Afterwards, rectangular glass frames, which had almost the same size as the area between the electrodes, were fixed with grease (High Vacuum Grease, Dow Corning, Wiesbaden, Germany) in the wells, enabling to lyse only treated cells by adding 200 μl cell lysing buffer (99.4 ml DMSO, 0.6 ml acetic acid (100%), 10 g SDS) into the glass frame. After 10 min of incubation, 100 μl of the lysate were pipetted into a 96-well plate and its absorbance was measured at 550 nm for a reference wavelength of 700 nm (Infinite M200 PRO, Tecan, Männedorf, CH). For a better comparison, not only PEF-treated but also PTM-treated as well as control cells were analyzed using the glass frames.

### Statistical analysis

Three independent experiments were performed, each by itself in triplicate, for each parameter set. Statistical significance was analyzed by a one-way ANOVA test together with Dunnett´s post-hoc test with significance levels associated with p-values of <0.05 (*), <0.01 (**) and <0.001 (***). (Since different dependencies were tested, significances are indicated by different symbols in the figures: *, #, ^.)

### Evaluation of electroporation by propidium iodide uptake

The formation of pores in the cell membrane due to the application of pulsed electric fields was analyzed by propidium iodide (PI) uptake. PI has a molecular weight of 670 Da and is therefore too large to permeate through an intact cell membrane. In the case of compromised membranes, such as for dead or electroporated cells, PI enters the cell and intercalates with DNA. This results in a fluorescence signal that can be detected. For the evaluation of the time course of possible electroporation, the medium on the cells was exchanged before and 5 min, 10 min, and 15 min after pulse exposure by complete medium, supplemented with 3 μM PI. Afterwards, cells were incubated for 10 min, rinsed twice with PBS and finally inspected under an inverted fluorescence microscope (Axio Observer D1, Carl Zeiss, Berlin, Germany).

## Results

### Cell viability after treatment with PTM and μsPEFs

**Fig 2:**
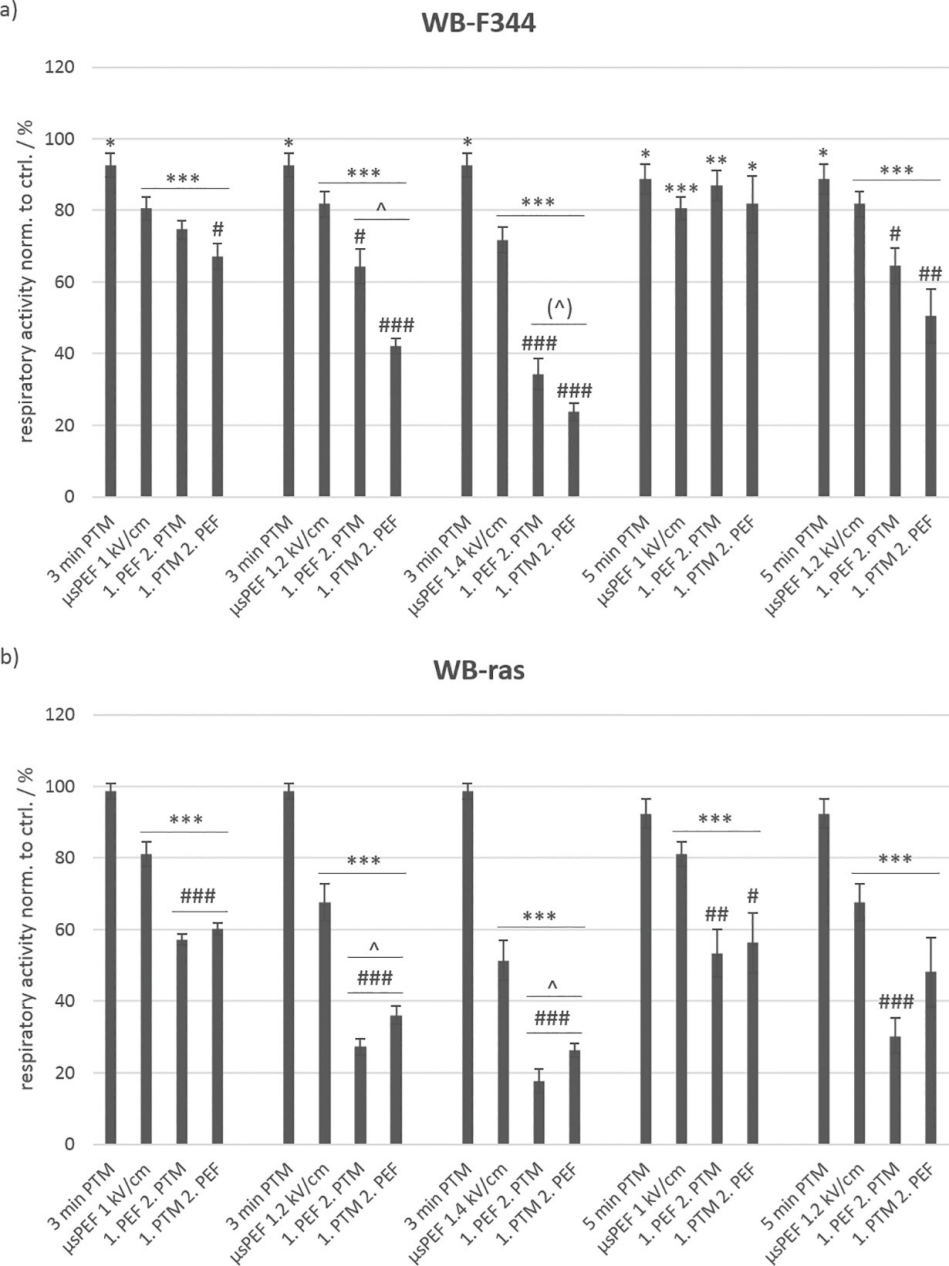
**Viability of non-tumorigenic WB-F344 (a) and tumorigenic WB-ras cells (b) after treatment with PTM, μsPEFs or a combination of both, determined 3 h after treatment.** The single treatments, i.e. with PTM or μsPEFs only, are compared to the combined treatments. Significant differences in cell viability are marked for the different treatment methods compared to control (*), for the combined treatments compared to the respective μsPEF-treatment alone (#) and between the two treatment orders (˄). Bars show mean values of three independent experiments performed in triplicates ± standard errors.

Cells were exposed either to cell culture medium that was previously treated with plasma for 3 min or 5 min (i.e. 3-min PTM or 5-min PTM) or to eight consecutive 100-μs pulses with field strengths of 1 kV/cm, 1.2 kV/cm and 1.4 kV/cm or to a combination of both methods. (All relevant abbreviations, especially treatment conditions, are summarized together with their respective explanations in S1 Table.) An MTT assay was performed, 3 h after treatments were concluded, to evaluate viability. Fig 2 shows the results for the viability of non-tumorigenic WB-F344 (a) and tumorigenic WB-ras cells (b).

Exposure to 3-min PTM and 5-min PTM alone resulted in only a minor decrease of cell viability, which was slightly significant against control (*) for WB-F344 cells but not for WB-ras cells. A longer plasma treatment time of the medium marginally increased the number of dead cells. In contrast, the application of μsPEFs alone significantly affected cell viability of both cell lines in comparison to controls. In case of WB-F344 cells, the fraction of dead cells (in comparison to control) increased from about 20% for a field strength of 1 kV/cm to about 30% for 1.4 kV/cm, and in case of WB-ras cells, from about 20% to about 50%, respectively.

The combined treatment of cells with μsPEFs and PTM clearly killed more cells than the single treatments alone. The lethality of the combined treatment compared to μsPEF exposures only was less pronounced for WB-F344 than for WB-ras cells. Significantly more tumorigenic WB-ras cells were killed with the combined treatment than with μsPEF treatments alone (Fig 2B: #). Viability decreased with increasing field strength from about 60% to about 26%, and fell even below 20% when μsPEFs were applied first. An increase of the plasma treatment time of the medium did not increase the number of dead cells for the combined treatment or single PTM-treatments considerably. However, there was a significant difference in cell survival for both cell lines depending on the treatment order, at least for the combination of 3-min PTM and μsPEFs with field strengths of 1.2 kV/cm and 1.4 kV/cm (Fig 2: ˄). Interestingly, while more of the tumorigenic WB-ras cells were killed when μsPEFs were applied first, the outcome was reversed for the non-tumorigenic WB-F344 cells and more cells died when PTM was applied first. (With a p-value of 0.0566, this difference was close to be significant for 3-min PTM in combination with μsPEFs of 1.4 kV/cm.)

**Fig 3:**
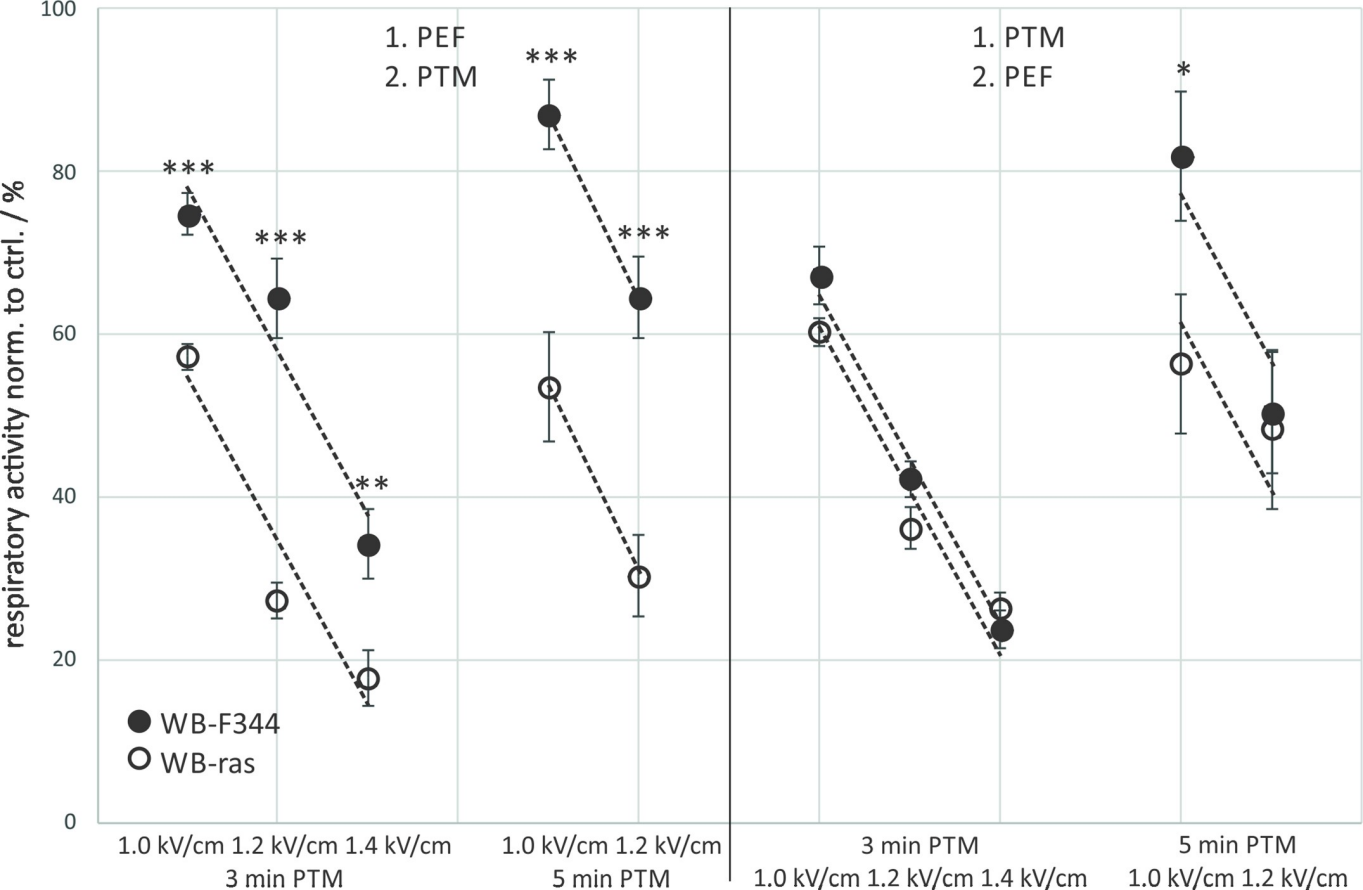
Comparison of the viability of WB-F344 (filled circles) and WB-ras cells (open circles) 3 h after combined treatment with PTM and μsPEFs, depending on the treatment order. Asterisks mark statistically significant differences in cell viability between the two cell lines for the treatment conditions that are described at the bottom of the graph. The graph shows mean values of three independent experiments performed in triplicates ± standard errors. Dashed lines indicate the rates at which respiratory activities decreased with increasing field strength.

A direct comparison of viabilities of WB-F344 and WB-ras cells with respect to the treatment order is shown in Fig 3. When μsPEFs were applied first, significantly more tumorigenic WB-ras cells died compared to non-tumorigenic WB-F344 cells for all exposure conditions (Fig 3: left frame). This difference was about 17% for the treatments with 3-min PTM combined with μsPEFs and about 35% for the combination with 5-min PTM. In contrast, when cells were exposed to PTM first, there was almost no difference in cell death between both cell lines (Fig 3: right frame). The dependency of respiratory activity on field strength was rather linear and the rates at which respiratory activity decreased with increasing field strength was approximately the same for both cell lines for the same exposure protocols grouped together (Fig 3: dashed lines).

**Fig 4:**
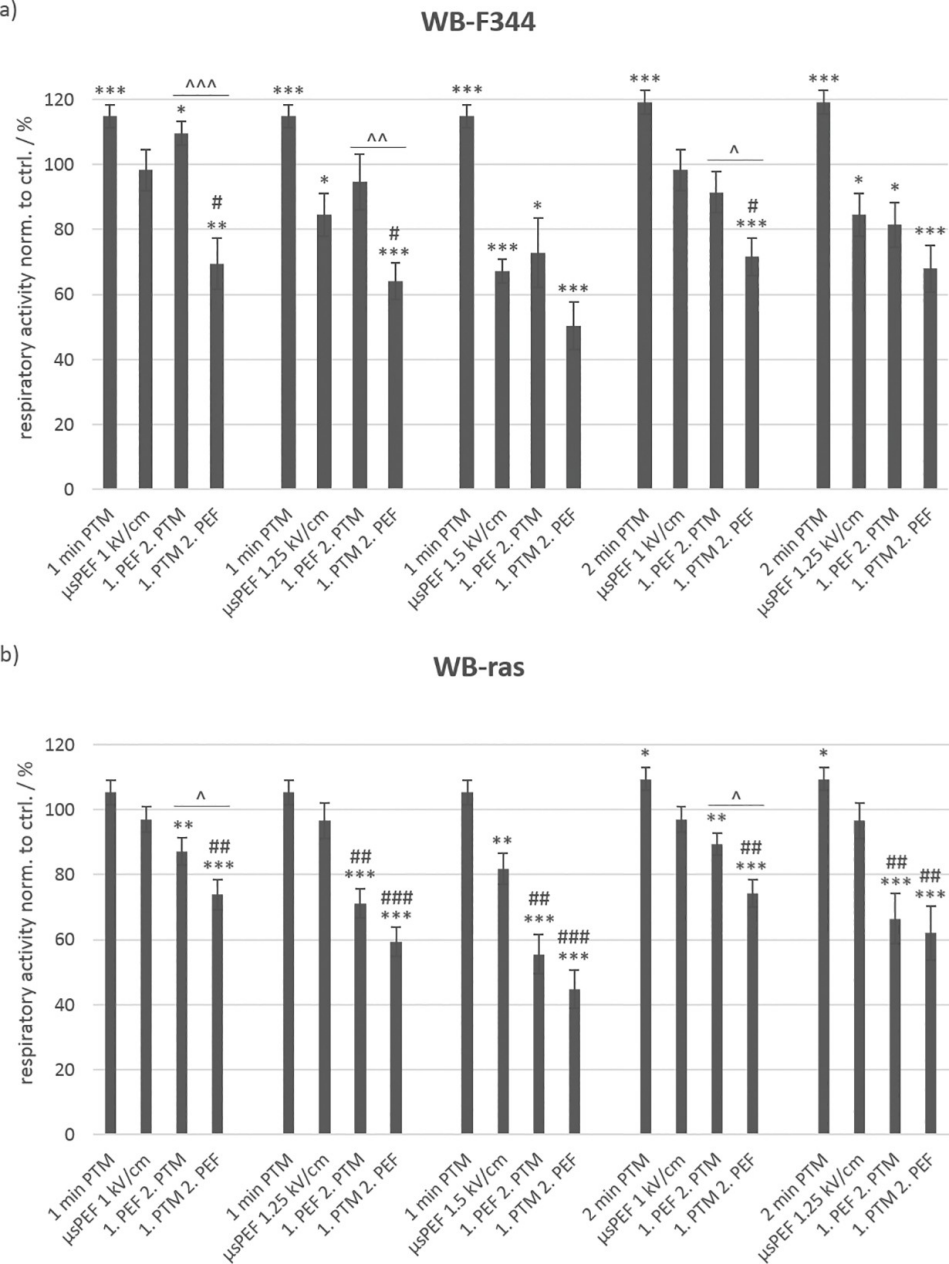
**Viability of non-tumorigenic WB-F344 (a) and tumorigenic WB-ras cells (b) after treatment with PTM, μsPEFs or a combination of both, determined 24 h after treatment.** The single treatments are compared to the combined treatments. Significant differences in cell viability are marked for the different treatment methods compared to control (*), for the combined treatments compared to the respective μsPEF-treatment alone (#) and for the two treatment orders (˄). Bars show mean values of three independent experiments performed in triplicates ± standard errors.

Since the combined treatment had a strong effect on cell viability but was rather independent from the plasma treatment times of the medium, cell survival was also determined for shorter plasma treatment times of 1 min and 2 min. Field strengths for exposures with μsPEFs were with 1 kV/cm, 1.25 kV/cm and 1.5 kV/cm in the same range as applied in previous experiments. The incubation time after treatment was extended to 24 h to account for a possible longer time that it might take PTM to exert its full impact on cells, e.g. until apoptotic processes have been induced. Results are shown in Fig 4 for non-tumorigenic WB-F344 (a) and tumorigenic WB-ras cells (b).

For these conditions, PTM-treatment alone had a stimulating effect on the WB-F344 cells with a cell respiratory activity that was significantly increased to about 120% compared to control. In addition, there was almost no difference if medium was plasma-treated for 1 min or 2 min. When cells were treated with μsPEFs alone, more cells died with increasing field strength, as was expected from previous experiments. While eight 100-μs pulses with an amplitude of 1 kV/cm had no significant effect on cell viability, only about 85% of the cells survived a field strength of 1.25 kV/cm and barely 70% a field strength of 1.5 kV/cm. The combined treatment with PTM and μsPEFs still showed an order-dependent effect on cell viability. When μsPEFs were applied first in combination with 1-min PTM, about 10% more cells survived the treatment compared to μsPEF-treatment alone. When PTM was administered first, cell viability decreased considerably. Significantly fewer WB-F344 cells, i.e. 40%, 30% and 20%, survived the combined treatment with 1-min PTM and μsPEFs of 1 kV/cm, 1.25 kV/cm and 1.5 kV/cm, respectively, compared to the combined treatment in reversed order.

In case of WB-ras cells, there was only a slight stimulating effect of PTM alone, which resulted in an increase of the cell respiratory activity by about 5% and 10% for 1-min PTM and 2-min PTM, respectively. Conversely, μsPEF-application alone had almost no effect on the viability of WB-ras cells. Only the exposure to pulses with a field strength of 1.5 kV/cm decreased cell viability by about 20%.

The combined treatment also showed different results for WB-ras cells compared to WB-F344. For all exposure conditions and independently from the treatment order, a significant drop in cell viability was found, whereby more cells died when PTM was applied first. The decrease in viability of WB-ras cells was linear with increasing field strength and fell to 55% and 44% when μsPEF or PTM was administered first, respectively. However, the effect was independent from the actual plasma treatment time of the medium of either 1 min or 2 min. The dependency of cell viability on the treatment order was not significant, except for the combination of μsPEFs of 1 kV/cm and 1-min PTM or 2-min PTM, which both resulted in a difference of about 15% between the treatment orders.

**Fig 5:**
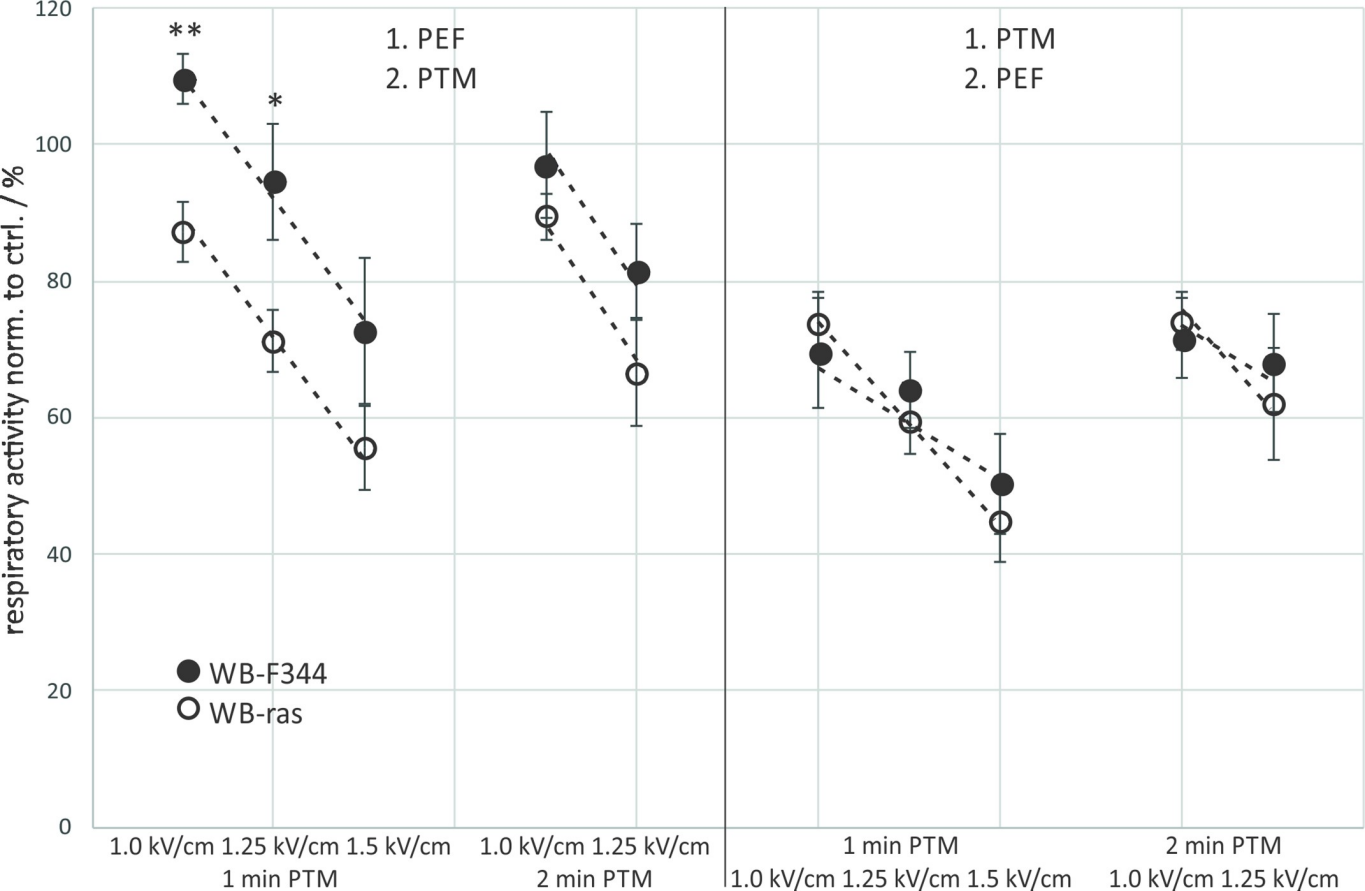
Comparison of the viability of WB-F344 (filled circles) and WB-ras cells (open circles) 24 h after combined treatment with PTM and μsPEFs, depending on the treatment order. Asterisks mark statistically significant differences in cell viability between the two cell lines for every treatment condition, which are described at the bottom of the graph. The graph shows mean values of three independent experiments performed in triplicates ± standard errors. Dashed lines indicate the rates at which respiratory activities decreased with increasing field strength.

When comparing cell viability of the two cell lines after combined treatment with respect to the treatment order, considerable differences could only be found when μsPEFs were applied first. The results were similar to those obtained for 3-min and 5-min PTM (Fig 3). The differences in cell viability were significant for 1 kV/cm and 1.25 kV/cm, followed by an exposure to 1-min PTM, resulting in an about 23% lower respiratory activity of WB-ras cells compared to WB-F344 cells (Fig 5: left frame). The combination of these field strengths with 2-min PTM resulted in a difference in viability between the two cell lines of about 12%. Similar to the previous experiments, viability decreased linearly with increasing field strength. The dependency of cell viability on field strength, represented by the slope of the dashed line, was roughly the same for 1-min and 2-min PTM for both cell lines, when μsPEFs were applied first. In contrast, when cells were exposed to PTM first, the effect on both cell lines was similar for all treatment options (Fig 5: right frame). The results further suggest that in this case the field strength-dependency of viability is less pronounced for WB-F344 cells than for WB-ras cells.

**Fig 6:**
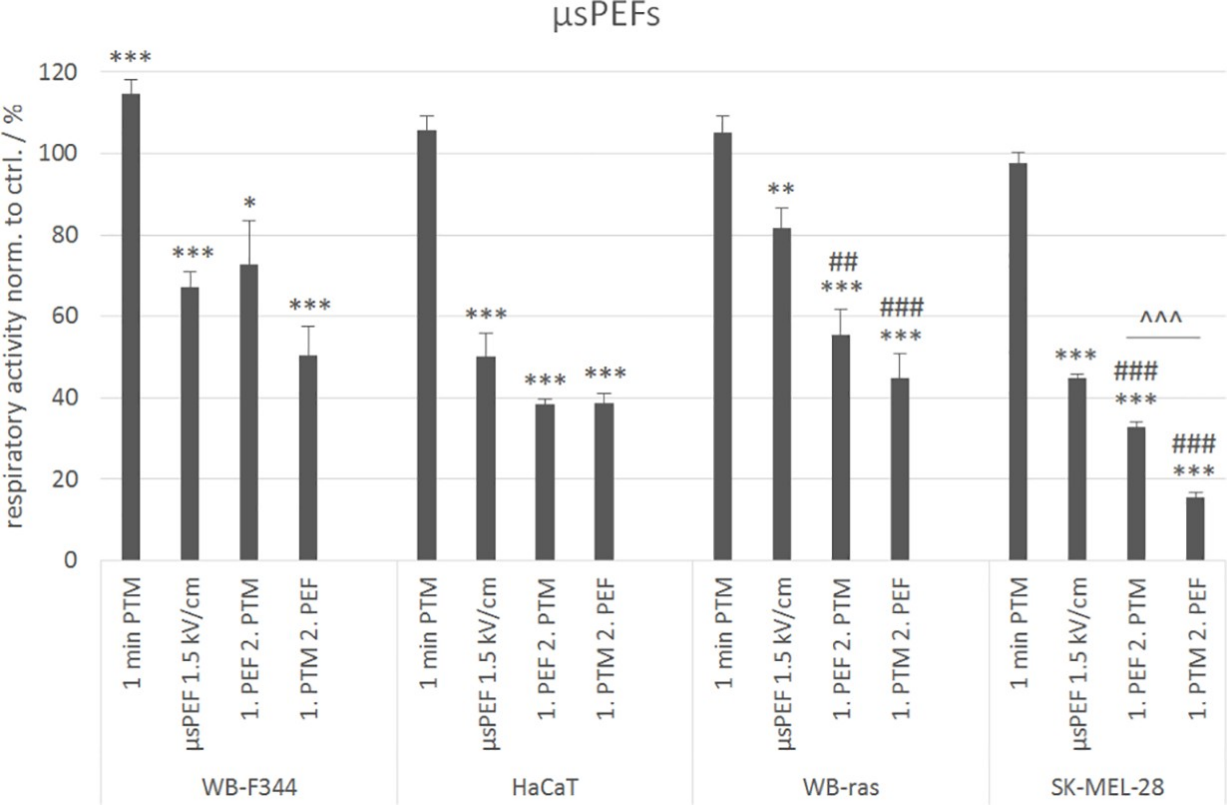
Comparison of the viabilities of WB-F344 and WB-ras cells with the viability of HaCaT and SK-MEL-28 cells after treatment with 1-min PTM, μsPEFs (1.5 kV/cm) or a combination of both, determined 24 h after exposure. Significant differences in cell viability are marked for the different treatment methods compared to control (*), for the combined treatments compared to the respective μsPEF-treatment alone (#) and for the two treatment orders (˄). Bars show mean values of three independent experiments performed in triplicates ± standard errors.

To verify effects of combination treatments also for other cell lines, experiments were repeated with monolayers of the human skin cell lines HaCaT and SK-MEL-28 with the best performing parameters that were determined for WB-F344 and WB-ras cells (1-min PTM, 8x100 μs pulses of 1.5 kV/cm, combination of both). The results for the comparison are shown in Fig 6.

HaCaT is an immortalized but non-tumorigenic cell line, and was chosen for the comparison with WB-F344 cells. In contrast, SK-MEL-28 is a tumorigenic, invasive cell line. Accordingly, this was compared with WB-ras cells. Fig 6 shows that the results for the human cell lines are similar to those obtained for the rat liver epithelial cells. The exposure to 1-min PTM had almost no effect on cell respiratory activity, while the application of μsPEFs alone significantly decreased cell respiratory activity to about half of the control-value for both human cell lines. The decrease of cell respiratory activity, compared to PEF treatment alone, after the combined treatment with μsPEFs and PTM was only significant for SK-MEL-28 cells, but not for HaCaT cells. For HaCaT cells, the treatment order did not seem to play a role. The cell respiratory activity decreased to about 40% compared to control in both cases. In contrast, a significant difference between the treatment orders for the combined treatment and also compared to μsPEF application alone was observed in case of the tumorigenic cell line SK-MEL-28. Cell respiratory activity decreased below 30%, when μsPEFs were applied first and fell to 15%, when cells were exposed to PTM first. Overall, the human cell lines seemed to be slightly more sensitive to the different treatments than the rat liver cell lines.

### Cell viability after treatment with PTM and nsPEFs

In order to investigate the importance of the pulse duration for combined treatments, monolayers were also exposed to 20 pulses of 100 ns with field strengths of 15 kV/cm, 20 kV/cm and 25 kV/cm. NsPEF application was again combined with exposures to 1-min PTM and 2-min PTM. The pulse parameters for nsPEFs were chosen to deliver about the same energy as the μs-pulses that were applied. An MTT assay was performed 24 h after treatment.

**Fig 7:**
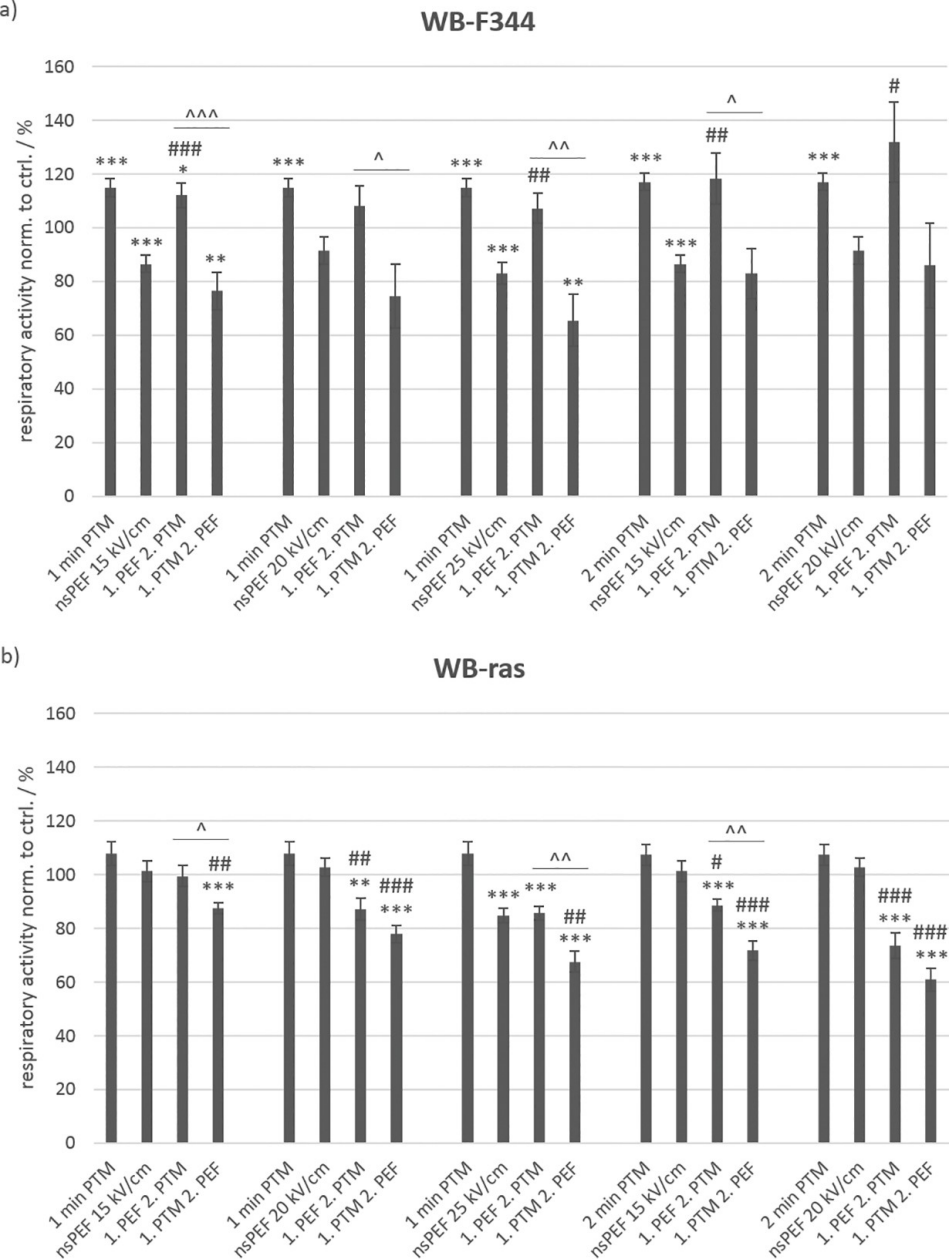
**Viability of non-tumorigenic WB-F344 (a) and tumorigenic WB-ras cells (b) after treatment with PTM, nsPEFs or a combination of both, determined 24 h after exposure.** The single treatments are compared to the combined treatments. Significant differences in cell viability are marked for the different treatment methods compared to control (*), for the combined treatments compared to the respective nsPEF-treatment alone (#) and for the two treatment orders (˄). Bars show mean values of three independent experiments performed in triplicates ± standard errors.

**Fig 8:**
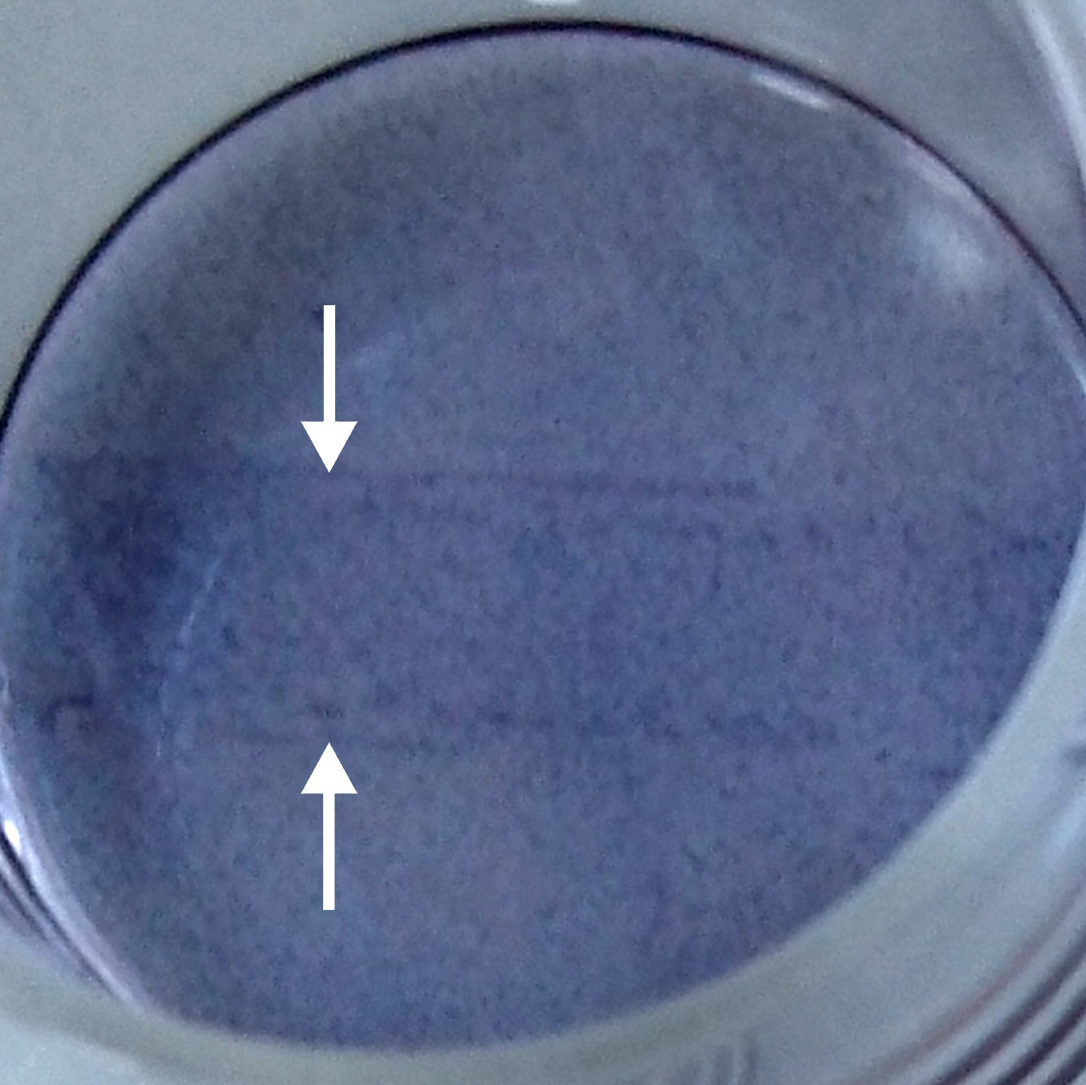
MTT assay of a WB-F344 monolayer that was treated first with 20 pulses of 100 ns and 20 kV/cm and subsequently with 2-min PTM. The picture was taken after an incubation time of 24 h. The area between the electrodes is clearly visible as a darker band in the middle of the well (white arrows point at previous electrode positions), indicating a stronger respiratory activity in this area.

The results obtained for the treatments with nsPEFs (Fig 7) were very similar to those obtained for μsPEFs (Fig 4). In case of WB-F344 cells (Fig 7A), 1-min PTM as well as 2-min PTM alone stimulated the respiratory activity significantly by about 20% compared to control. The application of nsPEFs alone decreased the viability by about 15%. However, an increase in field strength did not correspond to a similar loss in cell viability that was observed for an increase in field strength for μsPEFs. Moreover, the combined treatment with 1-min PTM, when nsPEFs were applied first, led to a stimulation of the cell respiratory activity by about 10% compared to control. Exposures to 2-min PTM after nsPEF-treatment with 15 kV/cm and 20 kV/cm resulted in an even more increased respiratory activity by about 20% and 30%, respectively. Fig 8 shows a WB-F344 monolayer 24 h after application of nsPEFs with 20 kV/cm and a subsequent exposure to 2-min PTM. The photograph was taken after incubation with the MTT-solution and before cell lysis. In the middle of the well, a darker band is clearly visible, representing the area between the electrodes (white arrows point at previous electrode positions). In agreement with the results presented in Fig 6A, the darker color indicates a stronger respiratory activity after the combined treatment in comparison to cells in the surrounding area, which were only exposed to PTM. Reversing the treatment order, i.e. administering PTM first, had a significant opposite effect on cell viability. For treatments with 1-min PTM, cell viability decreased with increasing field strength from 75% to 65%. The exposure to 2-min PTM combined with nsPEFs had no significant effect on cell viability, which was reduced to about 85% compared to controls for nsPEFs with 15 kV/cm and 20 kV/cm.

In case of WB-ras cells (Fig 7B), 1-min PTM and 2-min PTM alone had a slight but not significant stimulating effect on the cell respiratory activity. Exposures to nsPEFs alone did not affect cell viability, except for a field strength of 25 kV/cm, which resulted in a decrease of cell viability by about 15%. Regarding the combined treatment with 1-min PTM, when nsPEFs were applied first, 15 kV/cm had no effect on cell viability, while only about 85% of the cells survived 20 kV/cm and 25 kV/cm. In contrast, when cells were exposed to 1-min PTM first, cell viability decreased linearly from 87% to 67% with increasing field strength. An increase of the treatment time of the medium with plasma from 1 min to 2 min further increased the number of dead cells. Cell viability decreased by about 10% and 25% compared to controls, for 15 kV/cm and 20 kV/cm, respectively, when nsPEFs were applied first. When cells were exposed to PTM first, about 30% and 40% of the cells died.

**Fig 9:**
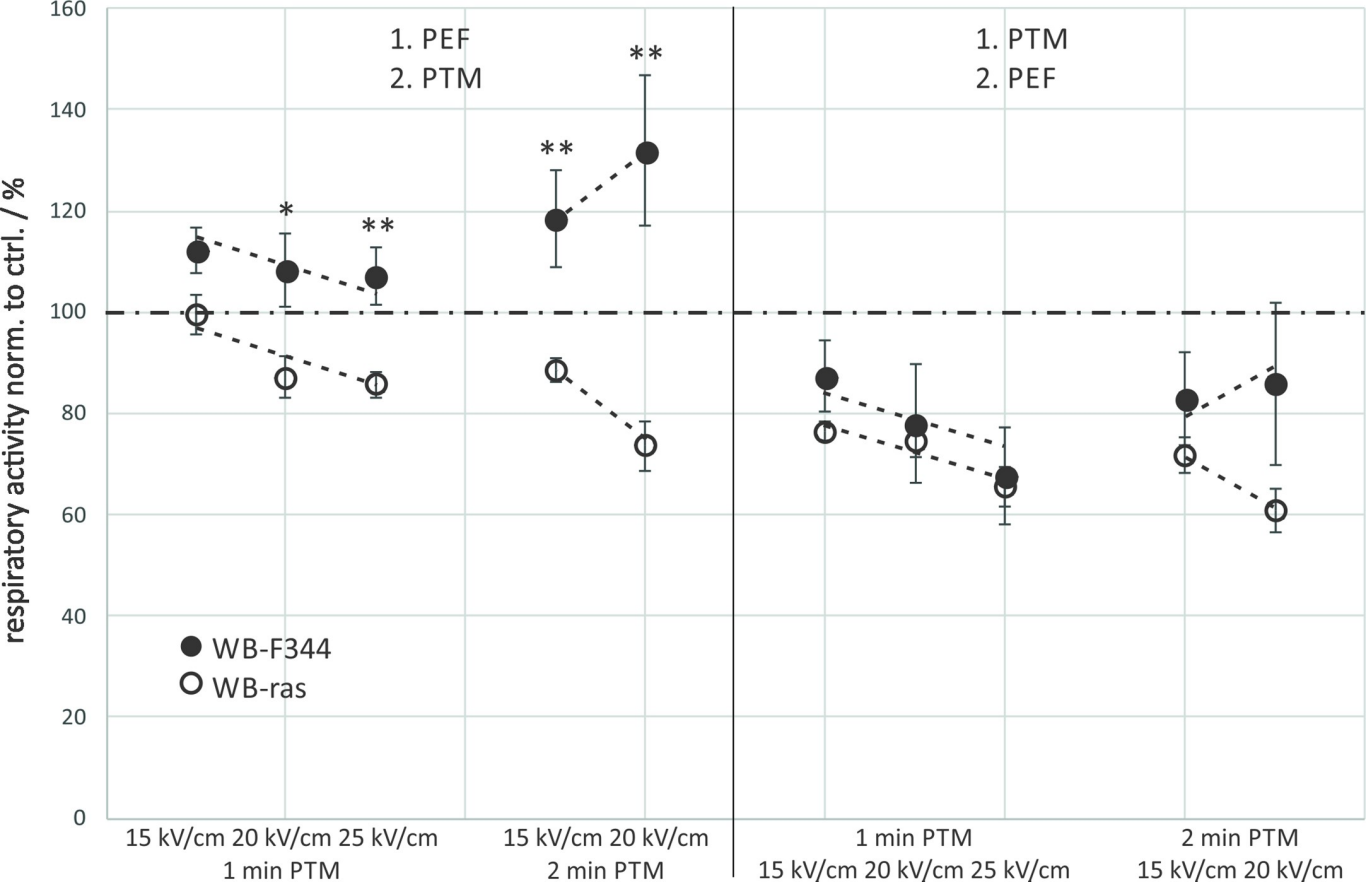
Comparison of the viability of WB-F344 (filled circles) and WB-ras cells (open circles) 24 h after combined treatment with PTM and nsPEFs, depending on the treatment order. Asterisks mark statistically significant differences in cell viability between the two cell lines for every treatment condition, which are described at the bottom of the graph. The graph shows mean values of three independent experiments performed in triplicates ± standard errors. Dashed lines indicate the rates at which respiratory activities decreased and increased, respectively, with increasing field strength. The dash-dotted line marks the control level.

The comparison of the cell respiratory activity of the two cell lines in dependency on the treatment order after combined treatments showed significant differences when nsPEFs were applied first (Fig 9: left frame). While the combined treatment for this order had a stimulating effect on non-tumorigenic WB-F344 cells, a killing effect was observed for tumorigenic WB-ras cells. In combination with 1-min PTM, the respiratory activity of both cell lines decreased with increasing field strength at about the same rate with respect to field strength. When combining nsPEFs with exposures to 2-min PTM, the effect was diverging, i.e. respiratory activity of WB-F344 cells increased with increasing field strength, while in case of WB-ras cells, respiratory activity decreased with increasing field strength. The difference in cell respiratory activity between the two cell lines for 2-min PTM, when nsPEFs were applied first, was rather large with 30% and 60% for 15 kV/cm and 20 kV/cm, respectively. In contrast, when cells were exposed to PTM first, viability of both cell lines was below control level for all treatment conditions. In this case, no significant differences in cell viabilities could be found for the two cell lines (Fig 9: right frame). Although respiratory activity dropped for all treatment conditions, notably, the respiratory activity of WB-F344 cells increased with increasing field strength for the combination with 2-min PTM, regardless of the treatment order.

**Fig 10:**
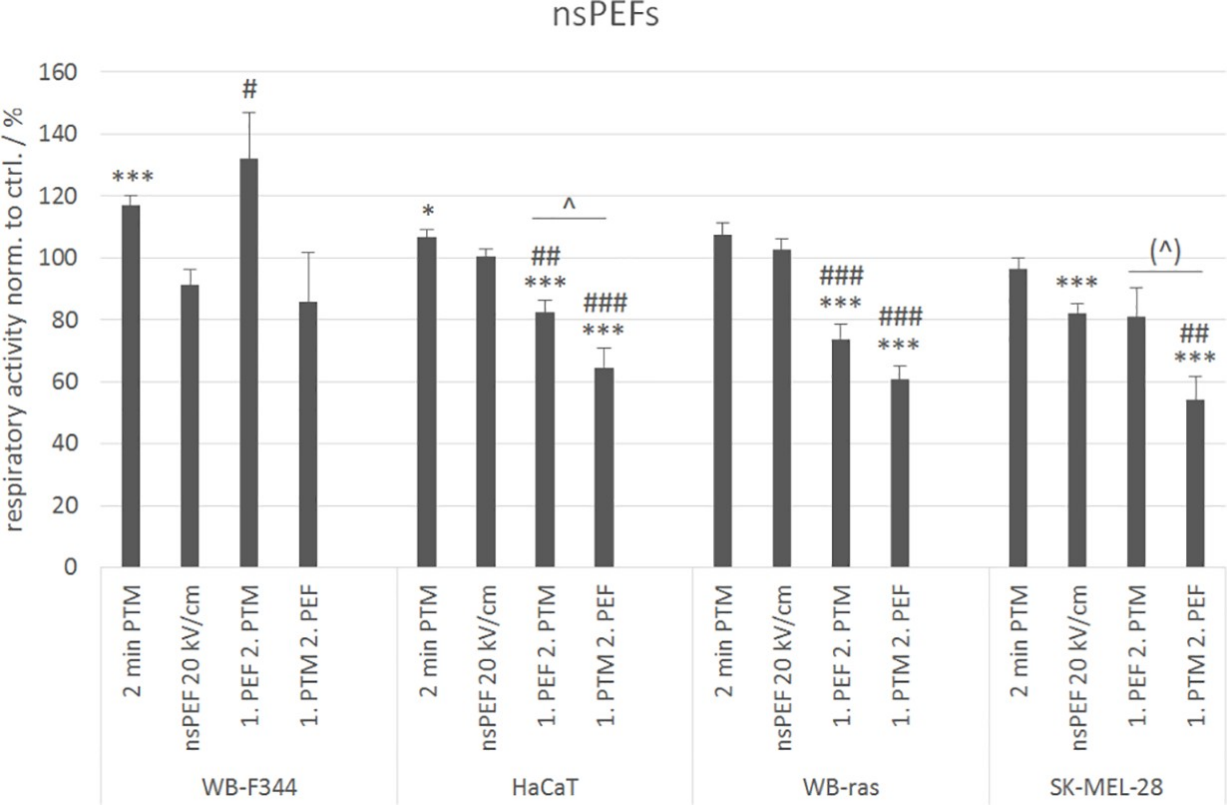
Comparison of the viabilities of WB-F344 and WB-ras cells with the viabilities of HaCaT and SK-MEL-28 cells after treatment with 2-min PTM, nsPEFs (20 kV/cm) or a combination of both, determined 24 h after exposure. The single treatments are compared to the combined treatments. Significant differences in cell viability are marked for the different treatment methods compared to control (*), for the combined treatments compared to the respective nsPEF-treatment alone (#) and for the two treatment orders (˄). Bars show mean values of three independent experiments performed in triplicates ± standard errors.

Experiments with 2-min PTM, 20 x 100-nsPEFs with 20 kV/cm or a combination of both were repeated with HaCaT and SK-MEL-28 cells to investigate if a stimulation or a decrease in cell respiratory activity can be found similar to WB-F344 and WB-ras cells (Fig 10).

The results obtained for the human cell lines were similar to those found for the rat liver epithelial cells. The only exception was, that no stimulation of the respiratory activity was detected after the combined treatment, neither for HaCaT nor for SK-MEL-28 cells. The exposure to 2-min PTM alone had a slightly stimulating effect on HaCaT cells but not on SK-MEL-28. In contrast, nsPEF application alone had almost no effect on HaCaT cells, but significantly decreased the cell respiratory activities of SK-MEL-28 cells by about 20% compared to control. Regarding the combined treatment, applying PTM first decreased cell respiratory activity more than applying PEFs first, while the reduction for the respective treatment in general was similar for both cell lines. Respiratory activity of both human cell lines was reduced to about 80% compared to control, when cells were exposed to nsPEFs first, and to about 64% for HaCaT and 54% for SK-MEL-28 when PTM was applied first. In contrast to the μsPEFs, the sensitivity of the human cell lines seemed to be comparable to that of the rat liver cells in case of the nsPEFs.

### Impact of sodium pyruvate in the cell culture medium

**Fig 11:**
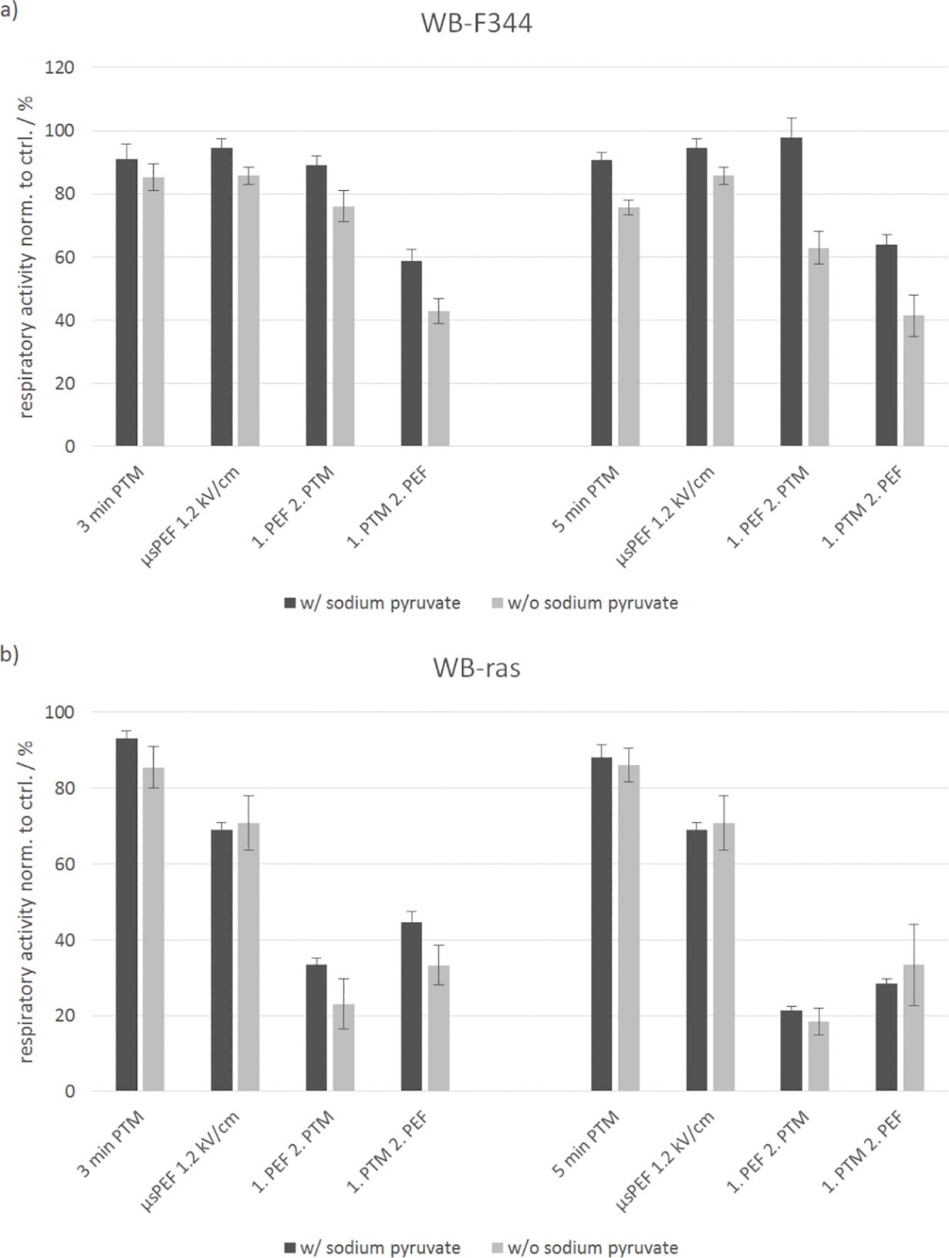
**Comparison of the viability of WB-F344 (a) and WB-ras cells (b) after treatment with 3-min and 5-min PTM, μsPEFs (1.2 kV/cm) or a combination of both in PTM with (dark grey) and without sodium pyruvate (light grey), determined 3 h after exposure.** The single treatments are compared to the combined treatments. Bars show mean values of two independent experiments performed in triplicates ± standard errors.

The results described above were all obtained from experiments conducted in cell culture medium containing sodium pyruvate, which is a known radical scavenger. Effects of PTM on cells are based on plasma-derived reactive species created in the medium. It is therefore conceivable that results for medium without sodium pyruvate might be different compared to medium with sodium pyruvate. To test this assumption, experiments on WB-F344 and WB-ras cells were repeated with 3-min and 5-min PTM and μsPEFs with 1.2 kV/cm, using DMEM with and without sodium pyruvate as basis for the PTM (Fig 11). Cell respiratory activity was determined 3 h after treatments.

For the non-tumorigenic WB-F344 cells, there was almost no difference in cell respiratory activity for exposures to 3-min PTM with and without sodium pyruvate. Respiratory activity decreased to 91% and 85%, respectively. In contrast, when exposed to 5-min PTM without sodium pyruvate, cell respiratory activity was about 15% lower compared to treatments with PTM containing sodium pyruvate. Respiratory activity decreased to 90% with sodium pyruvate and to 75% without sodium pyruvate. A slight difference, depending on addition of sodium pyruvate, of less than 10% could also be detected for μsPEFs application alone. With respect to the combined treatments, the difference in cell respiratory activity for medium with and without sodium pyruvate became more obvious and was more pronounced for experiments with 5-min PTM than with 3-min PTM.

In case of the non-tumorigenic WB-ras cells, the differences in cell respiratory activity were very weak for all exposure conditions. However, it should be noted that only two independent experiments were performed and that experiments should be repeated to obtain more reliable results.

### Electroporation by PEF-exposure

**Fig 12:**
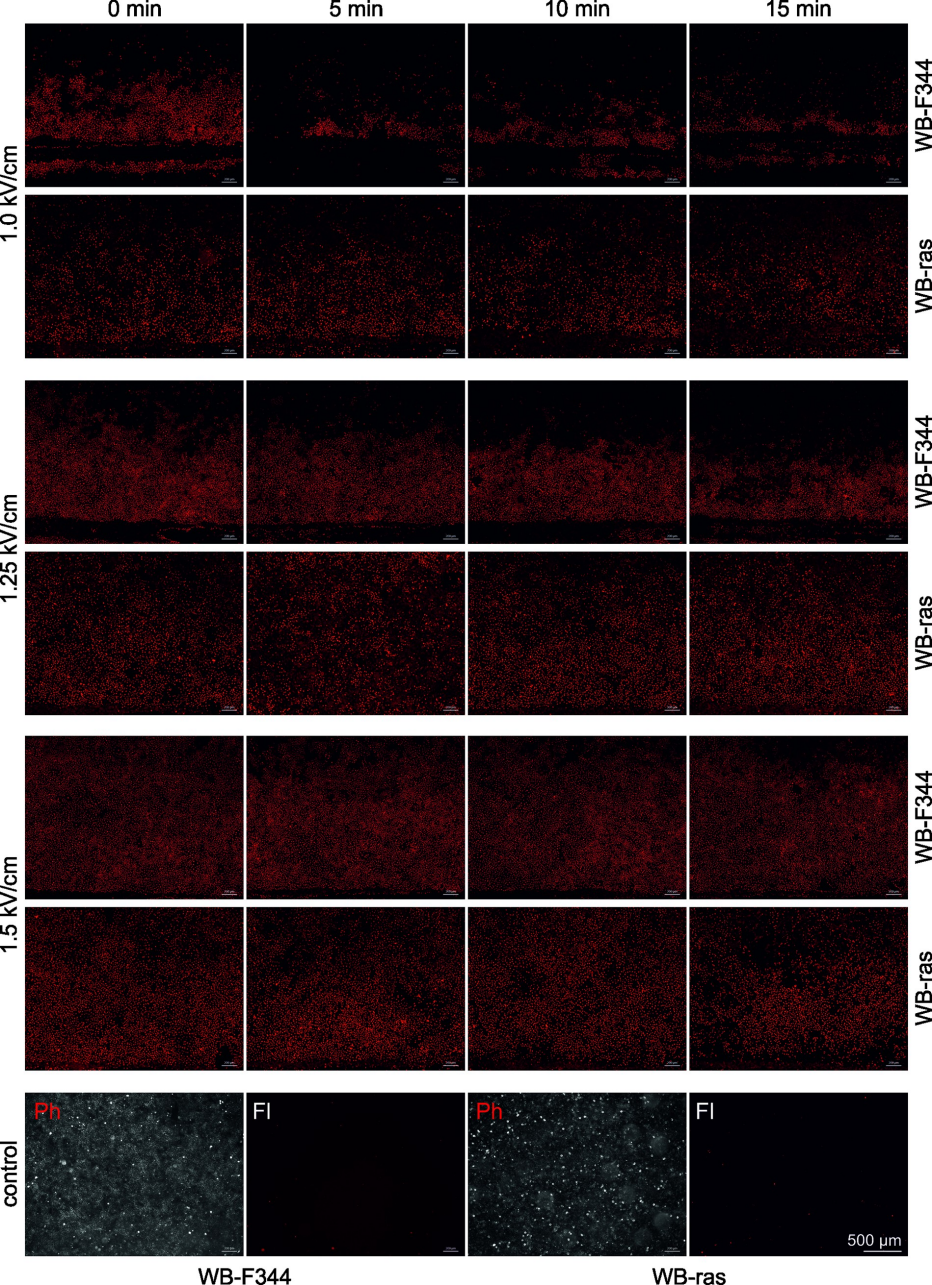
Electroporation indicated by propidium iodide (PI) uptake of WB-F344 and WB-ras monolayers, when PI was added before (0 min) and 5 min, 10 min and 15 min after exposure to 8 pulses of 100 μs with amplitudes of 1.0 kV/cm, 1.25 kV/cm and 1.5 kV/cm. Pictures were taken from the area close to one electrode at the bottom of each image. Note the scale bar in the last image. The bottom row shows phase contrast (Ph) and fluorescence images (Fl) of control monolayers.

Electroporation could facilitate the uptake of plasma-generated reactive species from the plasma-treated medium into the cells. Consequently, the formation of pores in the cell membrane of WB-F344 and WB-ras cells was determined by adding PI to the medium in the wells before and 5 min, 10 min and 15 min after the application of eight 100-μs pulses with field strengths of 1 kV/cm, 1.25 kV/cm and 1.5 kV/cm. In particular, resealing kinetics can be evaluated by adding PI at different time points after treatment. Pictures of the dye uptake were taken from the area close to one electrode (Fig 9). The electrode is shown either completely or partially as dark horizontal strip at the bottom of each image. The electric field is about 8% higher close to the electrodes compared to the center between the electrodes. A detailed description of the electric field distribution for the experimental setup was presented previously [47]. Field strength values indicated in Fig 12 refer to the electric field in the center between the electrodes. Since the electric field distribution of the applied pulses was not entirely homogenous, cells were not evenly electroporated over the entire treated area.

The number of electroporated cells was dependent on the actual field strength. The higher the pulse amplitude the more cells were electroporated. Moreover, electroporation efficacy was different for both cell lines. In case of WB-F344 cells, all cells closer to the electrodes and up to a certain distance were electroporated. For the lowest field strength of 1 kV/cm, all WB-F344 cells further away from the electrodes did not show electroporation. In contrast, for WB-ras cells, pore formation was also dependent on the distance from the electrode. Electroporation did also not appear to be as homogenous as for WB-F344 cells, i.e. for a given distance not all the cells showed PI-uptake. Furthermore, once electroporated, resealing of the pores was faster for WB-F344 than for WB-ras cells. This can in particular be seen for cells exposed to pulses with 1 kV/cm and 1.25 kV/cm. While PI-uptake of WB-ras cells looks similar for all time points, the amount of WB-F344 cells which took up the dye, decreased with time in dependency on the distance to the electrode. The cells which were furthest away from the electrodes resealed first.

The bottom row in Fig 12 shows phase contrast images of confluent monolayers of WB-F344 (left) and WB-ras (right) cells together with the corresponding fluorescence images. Almost no fluorescence signal could be detected for the controls.

**Fig 13:**
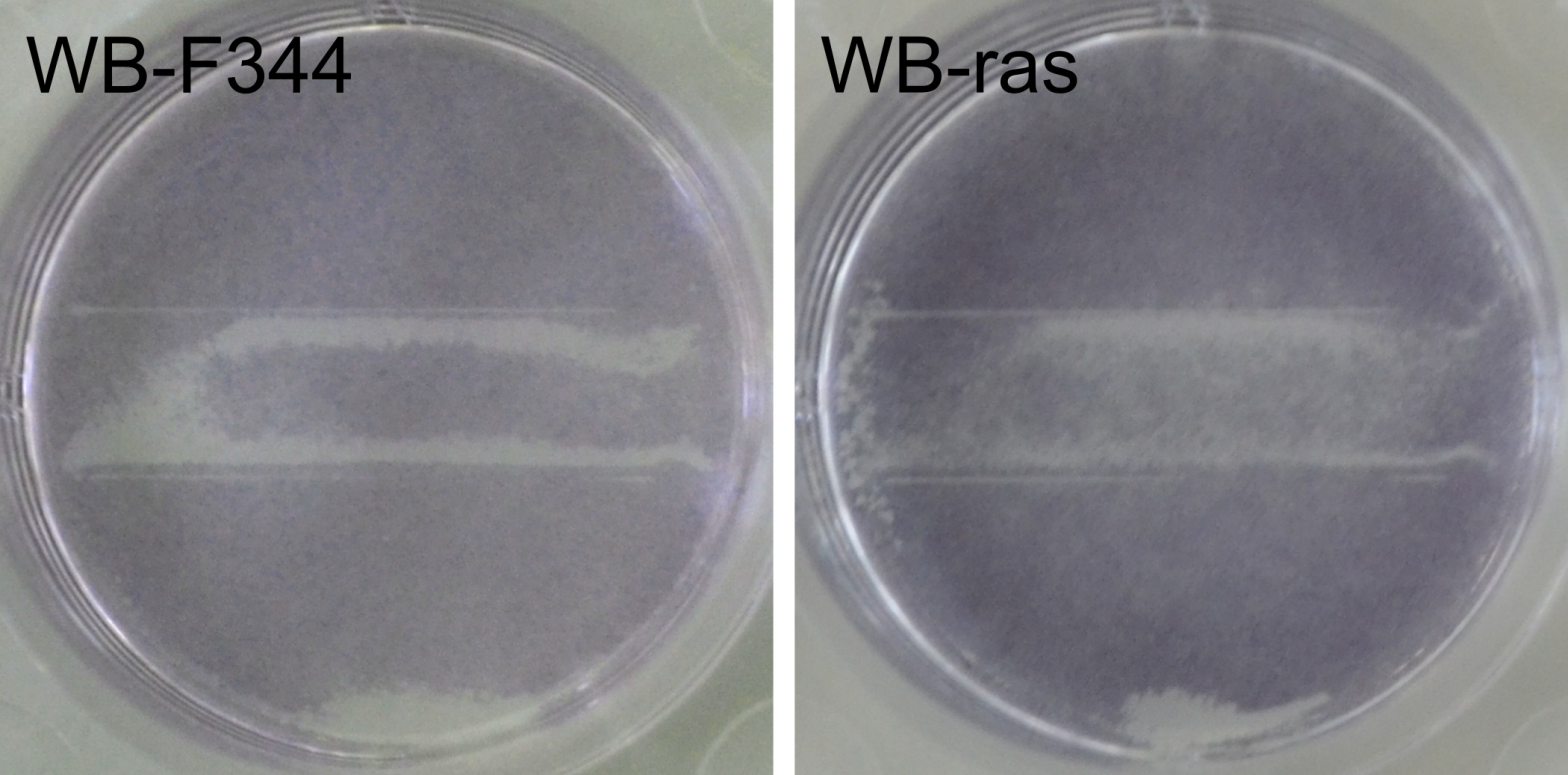
**Images of a WB-F344 (left) and a WB-ras monolayer (right) after application of 1-min PTM and subsequently eight 100-μs pulses with 1 kV/cm. Pictures were taken after incubation with MTT solution and before cell lysis**.

Fig 13 shows photographs of a WB-F344 and a WB-ras monolayer exposed to 1-min PTM first and afterwards to μsPEFs of 1 kV/cm. In case of WB-F344, only cells close to the electrodes did not form formazan. The cells that were located between the electrodes have the same color as the cells outside of the electrode area, indicating that the cells between the electrodes were not affected by the applied electrical pulses. The pattern is similar for the results obtained for PI-uptake. In contrast, WB-ras cells between the electrodes seem to be “paler” compared to the cells outside of the treated area, implicating that some of the cells were killed while some survived the treatment. In summary, results and distribution of effects for the MTT assay correlate with the results for electroporation obtained from PI-staining.

Cells were also investigated for PI-uptake for the same time points after exposure to 1-min and 2-min PTM. Fluorescence was measured by means of a plate reader (Infinite M200 PRO, Tecan, Männedorf, Switzerland), because no obvious increase in PI-uptake could be detected under the microscope. The results, which are presented in Table 1, support the conclusion that neither 1-min nor 2-min PTM induced electroporation.

**Table 1:** Electroporation indicated by propidium iodide (PI) uptake of WB-F344 and WB-ras monolayers, when PI was added before (0 min) and 5 min, 10 min and 15 min after exposure to 1-min and 2-min PTM. The values in the table represent the ratio of the fluorescence signal for each time point normalized to control (n = 1).

plasma treatment time of the medium	time after PTM exposure / min	fluorescence norm. to ctrl. / %	
		WB-F344	WB-ras
1 min	0	93.90	102.63
	5	101.39	96.28
	10	96.10	101.63
	15	107.12	97.15
2 min	0	106.67	99.52
	5	113.92	100.13
	10	96.51	99.36
	15	104.69	86.87

Electroporation by nsPEF was not tested, since in previous studies, no PI-uptake could be detected for 20 pulses of 100 ns with a field strength of 20 kV/cm, even when PI was added to the medium before nsPEF-application [47].

## Discussion

Possible mutual enhancing effects of plasma-treated cell culture medium in combination with microsecond and nanosecond pulsed electric fields on mammalian cells were investigated. While the effect of electrical pulses is primarily mediated by charging of cell membranes and subsequent pore formation, plasma acts mainly by generating highly reactive species in the liquid environment. Results of the MTT assay, obtained for the combined treatment, were compared to those of the respective single treatments. A summary of the results for all exposure conditions is shown in Table 2.

**Table 2:** Summary of MTT results.

**μsPEF-exposure: 8x100 μs, respiratory activity after 3 h incubation**						
	PTM	3 min	3 min	3 min	5 min	5 min
	PEF	1 kV/cm	1.2 kV/cm	1.4 kV/cm	1.0 kV/cm	1.2 kV/cm
WB-F344	1. PEF 2. PTM	-25%	-36%	-66%	-13%	-35%
	1. PTM 2. PEF	-33%	-58%	-76%	-18%	-50%
WB-ras	1. PEF 2. PTM	-43%	-73%	-82%	-47%	-70%
	1. PTM 2. PEF	-40%	-64%	-74%	-44%	-52%
	Single treatment	3 min PTM	5 min PTM	μsPEF 1.0 kV/cm	μsPEF 1.2 kV/cm	μsPEF 1.4 kV/cm
WB-F344		-7%	-11%	-19%	-18%	-28%
WB-ras		-1%	-8%	-19%	-32%	-49%
**μsPEF-exposure: 8x100 μs, respiratory activity after 24 h incubation**						
	PTM	1 min	1 min	1 min	2 min	2 min
	PEF	1 kV/cm	1.25 kV/cm	1.5 kV/cm	1.0 kV/cm	1.25 kV/cm
WB-F344	1. PEF 2. PTM	+10%	-5%	-27%	-9%	-19%
	1. PTM 2. PEF	-31%	-36%	-50%	-28%	-32%
WB-ras	1. PEF 2. PTM	-13%	-29%	-44%	-11%	-34%
	1. PTM 2. PEF	-26%	-41%	-55%	-26%	-38%
	Single treatment	1 min PTM	2 min PTM	μsPEF 1.0 kV/cm	μsPEF 1.25 kV/cm	μsPEF 1.5 kV/cm
WB-F344		+15%	+19%	-2%	-16%	-33%
WB-ras		+5%	+9%	-3%	-3%	-18%
**nsPEF-exposure: 20x100 ns, respiratory activity after 24 h incubation**						
	PTM	1 min	1 min	1 min	2 min	2 min
	PEF	15 kV/cm	20 kV/cm	25 kV/cm	15 kV/cm	20 kV/cm
WB-F344	1. PEF 2. PTM	+12%	+8%	+7% (#)	+18% (#)	+32% (#)
	1. PTM 2. PEF	-24%	-26%	-35%	-17%	-14%
WB-ras	1. PEF 2. PTM	-1%	-13%	-14%	-11%	-26%
	1. PTM 2. PEF	-13%	-22%	-32%	-28%	-39%
	Single treatment	1 min PTM	2 min PTM	nsPEF 15 kV/cm	nsPEF 20 kV/cm	nsPEF 25 kV/cm
WB-F344		+15%	+19%	-13%	-9%	-17%
WB-ras		+5%	+9%	+1%	+3%	-15%

Summary of MTT results obtained for all exposure conditions after incubation periods of 3 h and 24 h, respectively. The percentages describe mean values for the decrease (red labeled) or the increase (green labeled) of the respiratory activity after combined and single treatments in comparison to controls (100%). Values that are labeled with darker color indicate significant differences (p-value < 0.05) against control as well as against PEF-treatment alone. Lighter colors represent significant differences against control only (or against PEF-treatment alone, marked with #). Unlabeled results show no significant differences.

The results for the viability depending on the plasma treatment time of the medium, i.e. without additional exposure of the cells to PEFs, are consistent with previously reported studies [29–33]. Treatments with 3-min and 5-min PTM decreased cell viability, while 1-min and 2-min PTM had a rather stimulating effect on cell respiratory activity after an incubation time of 3 h and 24 h, respectively. Regarding cell killing, an increase of the plasma treatment time of the medium did not significantly increase the number of dead cells. This might be explained by the use of DMEM medium which contains sodium pyruvate as basis for the PTM. Sodium pyruvate is known for antioxidative effects. Adachi et al. found that when H_2_O_2_ was added to DMEM medium containing sodium pyruvate, the H_2_O_2_ concentration was decreased by 55% within 5 min [48]. An abundance of sodium pyruvate in the medium might therefore have resulted in similar concentrations of radicals that were generated by plasma exposure independent of the actual medium treatment times. Consequently, the concentration of reactive species in the medium was probably almost the same for medium treated with 1, 2, 3 or 5 minutes. However, the assumption that sodium pyruvate affects the results could not be conclusively decided. Comparing cell respiratory activity after treatment with medium containing sodium pyruvate or not, only the results for WB-F344 cells seemed to support this assumption. In general, more cells died when medium without sodium pyruvate was used compared to experiments with medium with sodium pyruvate. Without sodium pyruvate, the differences in respiratory activities were more pronounced for the longer plasma treatment time. In support of the hypothesis, a longer treatment time should result in a larger amount of reactive species that is not scavenged in medium without sodium pyruvate. In contrast, almost no differences in cell respiratory activity could be detected for WB-ras cells for experiments conducted with medium with or without sodium pyruvate. However, it should be noted that only two independent experiments were conducted to investigate the impact of sodium pyruvate on cell viability. Further experiments are needed.

The observed stimulating effect is more difficult to explain. Changes of concentrations of different reactive species in the plasma treated medium over time [49] might at least in part contribute to the observed treatment-time dependent effects. However, the qualitative and quantitative determination of reactive species in liquids after plasma treatment is very difficult, especially due to their short lifetime. Several studies using different plasma sources were performed to investigate the formation of reactive species in liquids. Several groups investigated the production of reactive species in DMEM cell culture medium and could determine different species, such as hydroxyl radicals, superoxide anion radicals, peroxynitrite, and singlet oxygen [48, 50, 51].

Both outcomes, killing as well as stimulation, were more pronounced for the non-tumorigenic WB-F344 cells than for the cancer cell line WB-ras. Interestingly, for PEFs (without PTM added), only a killing but not a real stimulating effect was observed, regardless of pulse duration and amplitude. This result confirms the crucial role of plasma-generated species for stimulation. Viability of WB-ras but not of WB-F344 was different after an incubation time of 3 h compared to 24 h for the treatment with μsPEFs. After 3 h, up to 50% of the WB-ras cells had not survived the PEF-treatment, while after an incubation time of 24 h, more than 80% of the WB-ras cells survived the exposure or had recovered from it. Since WB-F344 and WB-ras cells have a very similar proliferation time and migration velocity [52, 53], the difference cannot be explained by cells growing from the untreated area outside the electrodes into the area between the electrodes. Accordingly, viability should change in the same way for both cell lines. More likely, differences for both cell lines are due to transient changes of the cell respiratory activity, as determined by the metabolic assay. Cell respiratory activity of WB-ras cells was possibly transiently depleted, leading to a decreased formation of formazan. How μsPEFs are affecting the respiratory mechanisms in detail is not clear. In contrast, for WB-F344 cells, almost the same results were obtained for an incubation time of 3 h and 24 h after μsPEF-application, suggesting that in WB-ras cells other mechanisms are affected than in WB-F344 cells.

Exposure to nanosecond electric pulses led to similar cell death rates for WB-F344 and WB-ras cells, i.e. the distinct differences found for μsPEFs, especially in sensitivity, were not that pronounced for nsPEFs. However, for nsPEF-exposures, viability of WB-F344 cells was already decreased for a field strength of 15 kV/cm while WB-ras cells did not start to die unless 25 kV/cm were applied. Therefore, at least a field strength dependent sensitivity seems indicated.

The different responses of the two cell lines for the same exposure conditions might further be explained by possible differences in their membrane structures. Non-tumorigenic cells exhibit a different membrane composition than their tumorigenic counterpart, e.g. differences in membrane lipid contents, e.g. for cholesterol. These variations consequently also determine membrane fluidity, whereby no common pattern has been revealed [54]. The lipid composition as well as the fluidity of membranes in turn influence the efficacy of pore formation after PEF-exposure. This was already demonstrated for vesicles and cells [55, 56]. Consequently, one cell line might reach the threshold for electroporation faster and thus be more susceptible to cell death.

Another reason for different responses might be different cell shapes. WB-F344 cells look quite round under the microscope while WB-ras cells appear elongated. Consequently, electroporation of WB-ras cells depends more strongly on their orientation with respect to the applied electric field. This hypothesis was confirmed by results for PI-uptake (Fig 12). In WB-F344 cells, pore formation was dependent on the distance from the electrode due to the slightly inhomogeneous electric field, which was higher closer to the electrodes. In contrast, for WB-ras cells, electroporation was dependent on the distance to the electrode but not all cells with the same distance from the electrodes were electroporated. Accordingly, PI-uptake appears more “spotty”. However, WB-ras cells seemed to be electroporated more readily and pores seemed to reseal later than in WB-F344 cells, further supporting the hypothesis that WB-ras cells were more susceptible to the μsPEF-treatment than WB-F344 cells.

A few selected experiments were repeated with HaCaT and SK-MEL-28 cells to investigate if other, in particular human, cell lines respond to the different treatment conditions in the same way as it was observed for the rat liver epithelial cells. The results for the human cell lines were similar to those obtained for the rat liver cell lines. Human cell lines seemed to be a little more sensitive in comparison to the WB cells. This could be attributed to different cell characteristics (size, shape, lipid composition, etc.). Notably, in contrast to the WB-F344 cells treated with a combination of nsPEFs and PTM, no stimulation of cell respiratory activity could be found for HaCaT or for SK-MEL-28 cells. However, a direct comparison of HaCaT and SK-MEL-28 cells as a non-tumorigenic cell line and its tumorigenic counterpart is not appropriate, since both cell lines are not syngeneic. This was an inherent advantage of the comparison of WB-F344 and WB-ras cells. In general, it is expected that also other (human) cell lines respond in a comparable fashion to the combined treatment of PEFs and PTM, i.e. that both treatments can mutually reinforce their respective effects on cells. How cells react, meaning if they will be stimulated or if cell viability will be decreased, probably depends on the characteristics of the actual cell line.

Overall, the different mechanisms that are provided by the different treatment methods, especially for the killing of cells by PEF (e.g. electroporation, subcellular damage) and PTM (membrane oxidation, oxidative stress) encourage the combination of both methods to instigate a more profound response. Ideally, parameters can be chosen to achieve a distinct response with respect to selectivity. At least more effective treatment options can be provided.

The effect on cell viability after the combined treatment of cells with PTM and μsPEFs was dependent on the treatment order as well as on the cell line. Vernier et al. performed a molecular dynamics study showing that membranes containing oxidized lipids will be electroporated more readily than membranes without prior oxidative damage [57]. They could confirm their results with an *in vitro* experiment for peroxidized Jurkat cells. A significantly higher uptake of YO-PRO-1 was observed after exposure to 30-ns PEFs of 30 kV/cm in comparison to the treatment of non-oxidized cells. This result might explain the order-dependent differences of the combined treatment that was found in our study. The exposure of the cells to plasma-treated medium induces oxidative stress which might make cells more susceptible to PEFs. Another advantage of applying PTM first could be that reactive species are already present in the medium, when cells are exposed to PEFs. Consequently, the uptake of the species might by facilitated due to electroporation. A similar effect was already found for microorganisms by Zhang et al., who performed a study on the viability of *Staphylococcus aureus* after a combined treatment with plasma, which was applied directly to the bacteria suspension, and μsPEFs. They found a synergistic effect when bacteria were exposed to plasma first and an additive effect when PEFs were applied first. A transfer of reactive species into the bacteria and an acidification of the bacteria suspension were suggested as the reason for the synergistic effect. However, they do not give a detailed explanation for the dependency on the treatment order. In another study, Daeschlein et al. compared the efficiency of cold plasma, ECT and combined treatments in a melanoma mouse model [40]. They used the same plasma source kINPen 09 that we used for our study and the same treatment parameters. However, the plasma was applied directly to the tumor instead of preparing PTM. Only one plasma treatment time of 5 min, which was repeated daily for 5 days, combined with 2 x 8 electric pulses of 100 μs and 1 kV/cm was investigated. Without the addition of the cytostatic drug bleomycin, they did not find a significant effect in comparison to untreated controls. This might be explained by the low penetration depth of reactive species generated by the directly applied plasma, which is on the order of 25 μm for solid tissues [58, 59]. Consequently, the plasma would have induced only superficial alterations. In contrast, the penetration depth does not play a role for the investigated monolayers because every cell was exposed to PTM.

Regarding the plasma-derived reactive species, Jablonowski et al. hypothesize that the pathways of generation of reactive species in liquids after plasma treatment can, at least in part, be generalized, since hydrogen peroxide as well as nitrite and nitrate were found in all plasma-treated liquids, including water, (non-) buffered saline solution and cell culture media [49]. The presence of the stable end-product hydrogen peroxide in the liquid is taken as a general parameter for reactive oxygen species, whereas nitrite and nitrate represent reactive (oxygen and) nitrogen species. Furthermore, Jablonowski et al. state that the detected species do not differ generally, neither dependent on the working gas nor dependent on the treated liquid. They say that the quantity of the species differed dependent on the specific treatment conditions, but the detected stable end-products were more or less always the same. Accordingly, it can be assumed that species produced in the human body are at least very similar to those produced in the cell culture medium, although the quantity might differ.

In our experiments, all exposure conditions of the combined treatment killed more cells when PTM was applied first, with one exception. In case of WB-ras cells, results of the MTT assay, performed 3 h after treatment, showed less respiratory activity when μsPEFs were applied first. As indicated by the results for μsPEF-treatment alone, respiratory activity of the tumorigenic cells might have been transiently reduced, thus affecting the results after an incubation time of only 3 h, but not representing the real number of living cells.

The results of the MTT assay after an incubation time of 24 h were different for WB-F344 and WB-ras cells that were treated with 1-min PTM and 2-min PTM together with μsPEFs. In case of the non-tumorigenic WB-F344 cells, PTM treatment alone had a stimulating effect, while μsPEFs alone decreased the cell respiratory activity. When exposed to the combined treatment with μsPEFs applied first, the effects of PEFs and PTM seemed to cancel each other, meaning no significant reduction in viability compared to controls was observed. This cancellation effect was not found for WB-ras cells. Although PTM-exposure alone had a stimulating effect on the cells, the combined treatment resulted in a decreased viability of cells. The respiratory activity was actually significantly lower when cells were exposed to PTM first. The different responses of the two cell lines might again be explained by differences of the cell membrane composition and accordingly by different susceptibilities to the treatments. However, the exact mechanisms are still unknown.

In order to investigate the influence of the pulse duration on cell viability for combined treatments, in addition experiments were conducted with 100-ns pulses for which no pore formation could be detected by PI-uptake. Also in this case, in general more cells died when cells were exposed to PTM first. However, the response of the tumorigenic cell line was different from the response of the non-tumorigenic cell line, when nsPEFs were applied first. In case of WB-F344 cells, cell respiratory activity was increased for all exposure conditions for this treatment order. The combination with 2-min PTM increased the respiratory activity even above that of cells that were exposed to PTM alone. Instead of a cancellation effect, as found for μs-pulses, the treatment with ns-pulses and 2-min PTM seemed to induce a mutually reinforcement resulting in a stimulation. Conversely, for WB-ras cells, the combined treatments had a killing effect for all exposure conditions, which was more pronounced when PTM was applied first. In contrast to the results of the μs-pulse experiments, an increase of the plasma treatment time of the medium from 1 min to 2 min intensified both effects, i.e. the killing as well as the stimulating effect.

Differences in the results for the combined treatment with μs- and ns-pulses might be explained by the different pulse durations, which determine how cells are affected in detail. Exposures to ns-pulses penetrate cells and can in particular affect subcellular compartments and trigger associated biochemical pathways. In this case, plasma generated reactive species possibly interact with other biochemically relevant molecules and ions, such as calcium, that can be released from intracellular stores after nsPEFs-application [60]. Regarding μs-pulses, the ion concentration in the cell might be changed due to electroporation of the outer membrane resulting in a different plasma chemistry for the combined treatment than for PTM-exposure alone. However, mechanisms of combined treatments have yet to be investigated in more detail.

**Fig 14:**
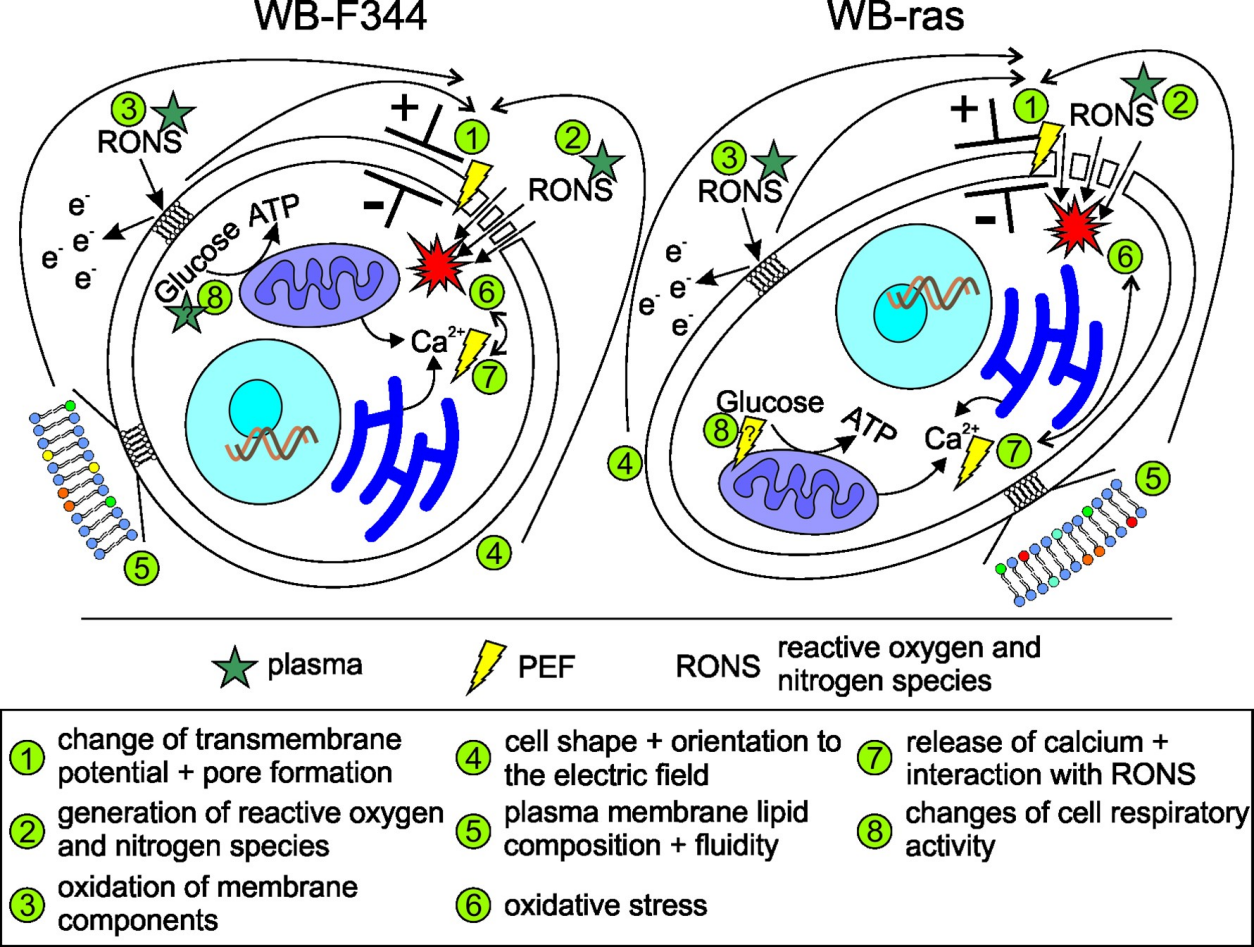
Possible (interaction) mechanisms of PTM and PEFs with cells. The exposure of cells to PEFs, in particular μsPEFs, can lead to a change of the transmembrane potential and subsequently to the formation of pores (1), enabling the entry of RONS, that are created in the plasma-treated environment (e.g. medium), into the cell (2). Oxidative species in the medium possibly peroxidize lipids of plasma membrane constituents (3), thus facilitating electroporation. The susceptibility of cells to PEFs furthermore depends on cell shape and diameter as well as the orientation of cells with respect to the applied electric field (4). In addition, the lipid composition and thus the fluidity of the plasma membrane affect pore formation (5). Intracellular concentrations of RONS lead to oxidative stress (6), which can cause DNA and protein damage. RONS can further react with other molecules, such as calcium, which can be released from intracellular stores by nsPEFs (7), forming other reactive species and enhancing the oxidative stress. Reactive species and maybe also PEFs may also change cell respiratory activity (8).

The possible interaction mechanisms between PTM and PEFs are summarized in Fig 14.

## Conclusion

For the first time a systematic study was conducted on the possibility to combine pulsed electric field and plasma treatments for the manipulation of mammalian cells. The combined application of PEFs and PTM on cells showed an enhanced response in comparison to the individual treatments. The outcome depended on the cell line as well as on the treatment order and the pulse duration. The combination of μsPEFs with PTM resulted in the killing of cells. The tumorigenic cell line WB-ras was more affected than its non-tumorigenic counterpart WB-F344. Furthermore, the effect was stronger, when cells were exposed to PTM first. In contrast, the combination of PTM with nsPEFs led to a stimulation of the respiratory activity of WB-F344 cells, when nsPEFs were applied first, while WB-ras cells were killed for both treatment order conditions. The results suggest that other mechanisms beyond simple pore formation play a crucial role for the cell´s response to the combined treatment. A combination of both methods could be tailored accordingly to improve the killing of tumor cells or the stimulation of cells, e.g. for wound healing.

## Supporting information

S1 Table List of symbols and treatment options and their meaning. (DOCX)
