# Supplementary material for: Cell stimulation versus cell death induced by sequential treatments with pulsed electric fields and cold atmospheric pressure plasma
Source: PLoS One. 2018 Oct 12;13(10):e0204916. doi: 10.1371/journal.pone.0204916 (PMC6193580; doi:10.1371/journal.pone.0204916)
Supplement: S1 Table — (DOCX) [file pone.0204916.s001.docx]

| **Symbols/treatments** | **Meaning** |
| --- | --- |
|  |  |
| **Significances** |  |
| * | Significant differences compared to control |
| # | Significant differences between combined treatment and PEF-treatment alone |
| ^ | Significant differences between treatment orders of the combined treatment |
| 1 symbol, e.g. * | p-value < 0.05 |
| 2 symbols, e.g. ** | p-value < 0.01 |
| 3 symbols, e.g. *** | p-value < 0.001 |
| Symbol in brackets | Almost significant, p-value close to (meaning slightly above) 0.05 |
|  |  |
| **Treatments** |  |
| 1 min PTM | Exposure of cell culture medium (DMEM) to plasma for 1 min, before it was applied to the monolayer |
| 2 min PTM | Exposure of cell culture medium (DMEM) to plasma for 2 min, before it was applied to the monolayer |
| 3 min PTM | Exposure of cell culture medium (DMEM) to plasma for 3 min, before it was applied to the monolayer |
| 5 min PTM | Exposure of cell culture medium (DMEM) to plasma for 5 min, before it was applied to the monolayer |
| µsPEF 1 kV/cm | Application of 8 x 100 µs-pulses with a field strength of 1.0 kV/cm |
| µsPEF 1.2 kV/cm | Application of 8 x 100 µs-pulses with a field strength of 1.2 kV/cm |
| µsPEF 1.25 kV/cm | Application of 8 x 100 µs-pulses with a field strength of 1.25 kV/cm |
| µsPEF 1.4 kV/cm | Application of 8 x 100 µs-pulses with a field strength of 1.4 kV/cm |
| µsPEF 1.5 kV/cm | Application of 8 x 100 µs-pulses with a field strength of 1.5 kV/cm |
| nsPEF 15 kV/cm | Application of 20 x 100 ns-pulses with a field strength of 15 kV/cm |
| nsPEF 20 kV/cm | Application of 20 x 100 ns-pulses with a field strength of 20 kV/cm |
| nsPEF 25 kV/cm | Application of 20 x 100 ns-pulses with a field strength of 25 kV/cm |
| 1. PEF 2. PTM | Treatment order: first, application of pulsed electric fields; second, application of plasma treated medium. Parameters for PEFs and PTM are the same as described for the single treatments that are compared in the respective graphs |
| 1. PTM 2. PEF | Treatment order: first, application of plasma treated medium; second, application of pulsed electric fields. Parameters for PEFs and PTM are the same as described for the single treatments that are compared in the respective graphs |
